# Skin dendritic cells in melanoma are key for successful checkpoint blockade therapy

**DOI:** 10.1136/jitc-2020-000832

**Published:** 2021-01-06

**Authors:** Anastasia Prokopi, Christoph H Tripp, Bart Tummers, Florian Hornsteiner, Sarah Spoeck, Jeremy Chase Crawford, Derek R Clements, Mirjana Efremova, Katharina Hutter, Lydia Bellmann, Giuseppe Cappellano, Bruno L Cadilha, Sebastian Kobold, Louis Boon, Daniela Ortner, Zlatko Trajanoski, Suzie Chen, Tanja D de Gruijl, Juliana Idoyaga, Douglas R Green, Patrizia Stoitzner

**Affiliations:** 1 Department of Dermatology, Venereology & Allergology, Medical University of Innsbruck, Innsbruck, Austria; 2 Department of Immunology, St. Jude Children's Research Hospital, Memphis, Tennessee, USA; 3 Department of Micobiology & Immunology, Stanford University School of Medicine, Stanford, California, USA; 4 Institute of Bioinformatics, Medical University of Innsbruck, Innsbruck, Austria; 5 Center of Integrated Protein Science Munich (CIPS-M) and Division of Clinical Pharmacology, Department of Medicine IV, Klinikum der Universität München, LMU Munich, Germany; 6 Member of the German Center for Lung Research (DZL), Munich, Germany; 7 German Center for Translational Cancer Research (DKTK), partner site Munich, Munich, Germany; 8 Bioceros BV, Utrecht, The Netherlands; 9 Ernest Mario School of Pharmacy and Rutgers Cancer Institute, Rutgers University, New Brunswick, New Jersey, USA; 10 Department of Medical Oncology, Cancer Center Amsterdam, Amsterdam UMC, Amsterdam, The Netherlands

**Keywords:** dendritic cells, immunotherapy, melanoma, tumor microenvironment, immunomodulation

## Abstract

**Background:**

Immunotherapy with checkpoint inhibitors has shown impressive results in patients with melanoma, but still many do not benefit from this line of treatment. A lack of tumor-infiltrating T cells is a common reason for therapy failure but also a loss of intratumoral dendritic cells (DCs) has been described.

**Methods:**

We used the transgenic tg(Grm1)EPv melanoma mouse strain that develops spontaneous, slow-growing tumors to perform immunological analysis during tumor progression. With flow cytometry, the frequencies of DCs and T cells at different tumor stages and the expression of the inhibitory molecules programmed cell death protein-1 (PD-1) and T-cell immunoglobulin and mucin-domain containing-3 (TIM-3) on T cells were analyzed. This was complemented with RNA-sequencing (RNA-seq) and real-time quantitative PCR (RT-qPCR) analysis to investigate the immune status of the tumors. To boost DC numbers and function, we administered Fms-related tyrosine 3 ligand (Flt3L) plus an adjuvant mix of polyI:C and anti-CD40. To enhance T cell function, we tested several checkpoint blockade antibodies. Immunological alterations were characterized in tumor and tumor-draining lymph nodes (LNs) by flow cytometry, CyTOF, microarray and RT-qPCR to understand how immune cells can control tumor growth. The specific role of migratory skin DCs was investigated by coculture of sorted DC subsets with melanoma-specific CD8+ T cells.

**Results:**

Our study revealed that tumor progression is characterized by upregulation of checkpoint molecules and a gradual loss of the dermal conventional DC (cDC) 2 subset. Monotherapy with checkpoint blockade could not restore antitumor immunity, whereas boosting DC numbers and activation increased tumor immunogenicity. This was reflected by higher numbers of activated cDC1 and cDC2 as well as CD4+ and CD8+ T cells in treated tumors. At the same time, the DC boost approach reinforced migratory dermal DC subsets to prime gp100-specific CD8+ T cells in tumor-draining LNs that expressed PD-1/TIM-3 and produced interferon γ (IFNγ)/tumor necrosis factor α (TNFα). As a consequence, the combination of the DC boost with antibodies against PD-1 and TIM-3 released the brake from T cells, leading to improved function within the tumors and delayed tumor growth.

**Conclusions:**

Our results set forth the importance of skin DC in cancer immunotherapy, and demonstrates that restoring DC function is key to enhancing tumor immunogenicity and subsequently responsiveness to checkpoint blockade therapy.

## Background

Invasive melanoma is the most fatal type of skin cancer.^1^ Although treatment with immune checkpoint inhibitors improves clinical outcome for patients with melanoma, there is still a large portion of patients that do not respond to this line of therapy.^2 3^ Resistance to checkpoint blockade therapy has been associated with decreased numbers of tumor-infiltrating effector T cells and increased numbers of immunosuppressive cells such as myeloid-derived suppressor cells (MDSCs) and regulatory CD4+ T cells (CD4+ Tregs). Moreover, infiltrating T cells are often impaired in their function.^4^

A recent study highlighted the importance of dendritic cell (DC)–T cell crosstalk during checkpoint blockade therapy.^5^ DCs are the most important antigen presenting cells for the induction of antitumor immune responses as they are equipped with the unique ability to cross-present exogenously derived antigen.^6–9^ It has been reported that DCs in patients with melanoma are decreased in their numbers, a fact that has been associated with a worse prognosis.^10^ DC numbers are normally kept under control by manifold mechanisms that mediate their survival and/or apoptosis.^11^ The various DC subsets in tissues are dependent on growth factors and cytokines. Langerhans cells (LCs), the only DC subset present in the epidermis, are very long lived cells and their homeostasis is dependent on transforming growth factor-β (TGF-β).^12 13^ Within the murine dermis, there are various DC subsets that can be distinguished on the basis of langerin, CD11b and CD103 expression; their survival is dependent on Flt3L.^14 15^ It has been reported that Flt3L can enrich all the conventional DC populations in various tissues except for LCs.^16–18^ Indeed, a recent study using melanoma mouse models has shown that treatment with Flt3L can increase intratumoral CD103+ cDC1 and that this subset has the most potent cross-presenting capacity in the tumor.^7^

The high percentage of patients with melanoma that do not respond to checkpoint blockade illustrates the need to develop combination approaches. For therapy development, preclinical studies that use mouse models that mimic human disease can be highly advantageous. In this study, we used the transgenic tg(Grm1)EPv spontaneous melanoma mouse model, in which melanocytes ectopically express the metabotropic glutamate receptor-1 (Grm1) under the control of the melanocyte-specific promoter dopachrome tautomerase.^19^ The same alteration has been observed in 40% of melanoma patient samples.^20^ The slow and continuous growth of melanoma lesions in these mice allows the investigation of immunological alterations over prolonged time at different tumor stages. We previously reported that during tumor progression, MDSCs infiltrate the tumor tissue at a late tumor stage and that they exert immunosuppressive effects.^21^ In this study, we investigated the immunological changes at earlier tumor stages and observed that melanoma development within the dermis was accompanied by upregulation of molecules that can inhibit T cell function and by the concomitant loss of the CD11b+ dermal cDC2 population. Our results show that tumor growth could be delayed when checkpoint blockade antibodies against programmed cell death protein-1 (PD-1) and T-cell immunoglobulin and mucin-domain containing-3 (TIM-3) were combined with a regimen that enhanced DC numbers and functionality, confirming the importance of DC for successful immunotherapy in melanoma.

## Materials and methods

### Mice

The tg(Grm1)EPv mice (kindly provided by Jürgen C. Becker, University of Duisburg-Essen) develop tumors predominantly in the ear and tail skin.^19 21^ Tg(Grm1)EPv mice were classified as tumor-free (TF) at the age of 6–10 weeks, and compared with tumor-early (TE, 4–6 months of age) and tumor-advanced (TA, 8–10 months of age) mice (representative images of mice from all stages are shown in online supplemental figure S1A). Breeding pairs for C57BL/6N mice were purchased from Charles River in Germany. Langerin-EGFP mice (kindly provided by Bernard Malissen, CIML, Marseille^22^) were crossed to the tg(Grm1)EPv mice to generate the tg(Grm1)EPv/langerin-EGFP mouse strain in which all Langerin+ DCs express EGFP. Pmel-1 mice with a transgene encoding for a CD8+ T cell receptor (TCR) recognizing the melanoma-associated antigen gp100^23^ were kindly provided by Thomas Tüting (Otto-von-Guericke University, Magdeburg). All mouse strains were housed and bred at the animal facility of the Department of Dermatology, Venereology and Allergology (Medical University of Innsbruck).

10.1136/jitc-2020-000832.supp1 Supplementary data

10.1136/jitc-2020-000832.supp2 Supplementary data

### Mouse experimental manipulations

Intratumoral injections into ear tumors were performed under anesthesia in a final volume of 25 µL per ear. Anesthesia was induced with intraperitoneal administration of a mix of PBS-Ketamine-Xylasol (ratio 1:1:2; aniMedica). Intraperitoneal injections were performed in a final volume of 100 µL. Ear thickness measurements for tumor growth monitoring in the tg(Grm1)EPv mice were conducted using a caliper (Kroeplin). A total of eight measurements per mouse were taken (four per ear) and from these the average ear thickness was calculated. Tumors in the tg(Grm1)EPv mouse model develop irregularly, and therefore, tumors of mice that are at the exact same stage can vary significantly. Thus, we displayed the ear thickness changes—Δear thickness—measured weekly. See online supplemental table 1 for reagents/antibodies used for in vivo treatments. For the transplantable melanoma experiments, C57BL/6N and tg(Grm1)EPv mice were injected subcutaneously into flank skin with 1.5×10^5^ B16.OVA cells (kindly provided by Franca Ronchese, The Malaghan Institute of Medical Research, New Zealand) in PBS (Phospate buffered saline). Tumor growth was measured with a caliper and calculated by length×width.

10.1136/jitc-2020-000832.supp3 Supplementary data

### Cell culture

For in vitro cell culture, we used RPMI medium (Lonza) supplemented with 10% heat-inactivated fetal calf serum (FCS, PAN-Biotech), 50 Units/mL penicillin/streptomycin (Life Technologies) and 2 mM L-glutamine (Lonza).

### Flow cytometry analyses of mouse skin, tumors and lymph nodes

Ear skin/tumors/LNs of tg(Grm1)EPv and C57BL/6N mice were processed as described previously.^21^ All staining steps for flow cytometry were performed for 15 min at 4°C, unless stated otherwise. Non-specific Fc-receptor-mediated antibody binding was blocked with incubation for 15 min with anti-CD16/32 antibody (clone 2.4G2, TONBO Biosciences). For exclusion of dead cells, samples were stained with eFluor-780 fixable viability dye (eBioscience), prior to any other staining step. Surface staining for CCR7 was performed for 30 min at 37°C. Restimulation of LN cell suspensions for intracellular cytokine staining was performed for 40 hours at 37°C with plate-bound anti-CD3 (5 µg/mL, clone 17A2, BD Biosciences) and soluble anti-CD28 (1 µg/mL, clone 37.51, BD Biosciences). Tumor cell suspensions were restimulated with Dynabeads Mouse T-activator CD3/CD28 (Gibco) in a ratio of 1:1 beads:total cells, overnight at 37°C and were supplemented with 30 Units/mL of recombinant mouse IL-2 (Peprotech). Cytokine release was blocked by addition of Brefeldin A (eBioscience) 4 hours prior to staining. For intracellular staining, the cells were fixed and permeabilized using the BD Biosciences Cytofix/Cytoperm Kit according to the manufacturer’s protocol. For FoxP3 staining, the cells were fixed overnight and permeabilized using the FoxP3 staining buffer kit according to the manufacturer’s protocol (eBioscience). For detection of gp100-specific CD8+ T cells we used the H-2Db restricted KVPRNQDWL pentamer (Proimmune) according to the manufacturer’s protocol. All analyses were performed on a FACS Canto II (BD Biosciences) and CytoFLEX S (Beckman Coulter Life Sciences). See antibody list in online supplemental table 2.

### Mass cytometry/CyTOF

Metal-labeled antibodies were obtained from Fluidigm or labeled in-house using the MaxPar X8 labeling kit (Fluidigm) according to the manufacturer’s instructions.^24^ Barcoding of samples was done with four different anti-CD45 metal conjugated antibodies (Y89 and palladium-labeled [Pd104, Pd106, and Pd108]). Immune cells were enriched using a density gradient of 40/90% Percoll (GE Healthcare). Staining was done as previously described.^24^ In brief, Fc receptors were blocked using anti-CD16/CD32 antibodies and cells were stained with 1 mL cisplatin (0.25 µM in PBS; Fluidigm) for 5 min at room temperature (RT) to exclude dead cells. Cells were stained with anti-CD45 barcoding antibodies for 30 min on ice. Samples were pooled for surface staining and incubated with the heavy metal conjugated antibody cocktail for 20 min on ice. Cells were placed in 2% paraformaldehyde overnight at 4°C. The following day, cells were resuspended with permeabilization buffer (eBioscience/Invitrogen) for 30 min on ice and incubated at RT in 125 nM iridium intercalator (Fluidigm). Cells were washed with water, filtered, and acquired with the CyTOF2 (Fluidigm) at the Stanford Shared FACS Facility (Stanford University, USA). For CyTOF data analysis, FCS-formatted files were first normalized with Premessa (https://github.com/ParkerICI/premessa/). Live, single cells, CD3- CD19- CD335- CD127- Ly6G- and CD45+ were gated using FlowJo. Events of interest were imported into CYT and transformed using arcsin (asinh x/5).

### Isolation of CD8+ T cells, CFSE labeling and in vitro cocultures

CD8+ T cells were isolated from pmel-1 TCR transgenic mice recognizing the gp100 antigen.^23^ LNs and spleens underwent enzymatic digestion as described before.^21^ CD8+ T cells were isolated using the mouse CD8a+ T cell isolation kit from Miltenyi according to the manufacturer’s instructions. For proliferation assays, isolated CD8+ T cells were labeled with 0.4 µM carboxyfluorescein succinimidyl ester (CFSE, Thermo Fisher Scientific) in PBS for 3 min at RT. For isolation of migratory skin DC subsets, tumor-draining LNs of 7 tg(Grm1)EPv/Langerin-EGFP mice were isolated and enzymatically digested as described before.^21^ CD45+ cells were enriched with a 1.119 g/mL density gradient according to the manufacturer’s protocol (Lymphoprep, Alere Technologies). Cell suspensions were then stained and sorted with a BD FACSAria II (BD Biosciences) (gating strategy described in online supplemental figure S4C). Equal numbers of the various DC subsets were cocultured in a ratio of 1:3 with T cells (3×10^5^ DC:9×10^5^ T cells) for 3 days in the presence of 50 Units/mL recombinant mouse IL-2 (Peprotech).

10.1136/jitc-2020-000832.supp4 Supplementary data

### CXCL9 and CXCL10 quantification in tumor lysates and blood serum

To determine the expression of the chemokines CXCL9 and CXCL10, tumors were homogenized and resuspended in lysis buffer (BioRad Laboratories). Protein concentrations were determined by Bradford assay (BioRad Laboratories). All samples were diluted to a protein concentration of 5 mg/mL and CXCL9/CXCL10 concentrations were analyzed by ELISA (R&D Systems, CXCL9 Cat#DY492 and CXCL10 Cat#DY466). Absorbance was measured with a Mithras LB 940 Multimode Microplate reader (Software MicroWin 2000), and concentrations were calculated as pg of cytokine per mg protein. Serum was analyzed without further dilution and CXCL9/CXCL10 levels were calculated as pg per mL serum.

### RNA isolation

For RT-qPCR and for RNA-seq, RNA from mouse skin and tumors was isolated using TRIzol Reagent (Life Technologies) according to the manufacturer’s instructions. For microarray expression assays, RNA was isolated using the RNeasy Mini kit (Qiagen) according to the manufacturer’s instructions.

### Real-time quantitative PCR

For RT-qPCR, genomic DNA was removed from total RNA with the RapidOut DNA removal kit (Thermo Fisher Scientific) and was reverse-transcribed into cDNA with random hexamers and SuperScriptR II Reverse Transcriptase (Life Technologies) according to the kit’s instructions. qPCR analysis was performed on a BioRad CFX96 using Brilliant III Ultra-Fast qPCR and RT-qPCR Master Mix (Agilent technologies). Sequences for probes and primers specific for mouse TATA-binding protein were selected by Primer Express software (Applied Biosystems) and synthesized by Microsynth. See online supplemental table 3 for the primers used.

### RNA-sequencing analysis

Library preparations and RNA-seq was performed at the Medical University of Innsbruck Sequencing Core Facility according to the following procedure. Total RNA was extracted with Trizol (see above), quality validated with the Agilent Bioanalyzer and submitted to QuantSeq 3′ mRNA-Seq library preparation, following the manufacturer’s instructions (Lexogen). Resulting libraries were sequenced with the Ion Proton System (Thermo Fisher Scientific). The fastq reads were first processed though quality control pipeline consisting of 3′ adapter removal with Cutadapt^25^ and quality trimming with Trimmomatic^26^ to remove bases with bad quality scores. All reads shorter than 22 nucleotides were removed. The quality trimmed reads were then mapped to the *Mus musculus* mm10 genome using a two-step alignment method; first alignment with STAR,^27^ followed by alignment of the unmapped reads using Bowtie 2.^28^ From the reads that mapped to multiple locations in the genome, only the primary alignment was retained. Reads that mapped to ribosomal RNA locations in the genome were removed from further analysis using the *split_bam.py* script from the quality control package RSeQC.^29^ HTSeq-count^30^ was used to count how many reads map to each gene in an annotation file. The DESeq2^31^ R package was used to test for differential expression. The p values were adjusted for multiple testing based on the false discovery rate using the Benjamini–Hochberg approach. Analysis and visualization of Gene Ontology terms associated with differentially expressed genes was performed using g:Profiler.^32^ Both groups of genes (up and downregulated, q value <0.1) were used as dual input for GO analysis. The biological terms are grouped together based on their shared genes where the similarity between terms is calculated using kappa statistics. The most significant term was chosen as a representative of the group (Benjamini–Hochberg correction).

### Microarray

Total RNA was isolated using the RNeasy Mini kit (Qiagen), according to the instructions of the manufacturer. The quality of the extracted RNA was evaluated by visualizing the ribosomal peaks on the Agilent Bioanalyzer 2100 and concentration was determined by the Nanodrop 8000. The samples were run on the Clariom S mouse arrays from Affymetrix/ThermoFisher utilizing the Affymetrix Whole Transcript Plus protocol which starts with 100 ng of total RNA as input. The final concentration of fragmented, biotin labeled ss-cDNA added to the hybridization mix which went onto the array was 2.3 µg. The arrays were then hybridized at 45°C while rotating at 65 rpm for 16 hours. The Clariom S mouse arrays were then washed and stained with the appropriate protocol for the Affymetrix Fluidics Station 450 and then was scanned on the Affymetrix GeneChip Scanner 3000 7G. The generated CEL files were analyzed in R using the *oligo*^33^ package and annotated using the *annotation*^34^ package with the clariomsmousetranscriptcluster^35^ database. Differential expression of probes was determined using the *limma*^36^ package, and heatmaps were generated using superheat. Pathway analyses were conducted on the raw probe data using gene set enrichment analysis (GSEA).^37^

### Statistical analysis

To determine whether parametric or non-parametric statistical tests should be used, all datasets from flow cytometry and RT-qPCR were tested for normality using the D’ Agostino-Pearson test. For up to two groups, statistical significance was determined by two-tailed unpaired Student’s t-test (parametric) or Mann-Whitney test (non-parametric). For more than one group, statistical significance was determined with one-way analysis of variance (ANOVA) followed by Tukey’s multiple comparison test (parametric) or Kruskal-Wallis test followed by Dunn’s multiple comparison test (non-parametric). For repeated measurements within one experiment, Friedman test (non-parametric) or repeated measures one-way ANOVA (parametric) was used. A p value of <0.05 was considered statistically significant (*), <0.01 very significant (**), <0.001 highly significant (***) and <0.0001 extremely significant (****). Error bars represent SE of the mean. All statistical analyses and graphics were performed using Graphpad Prism V.8.0.

## Results

### Immunosuppressive molecules increase with tumor progression

**Figure 1:**
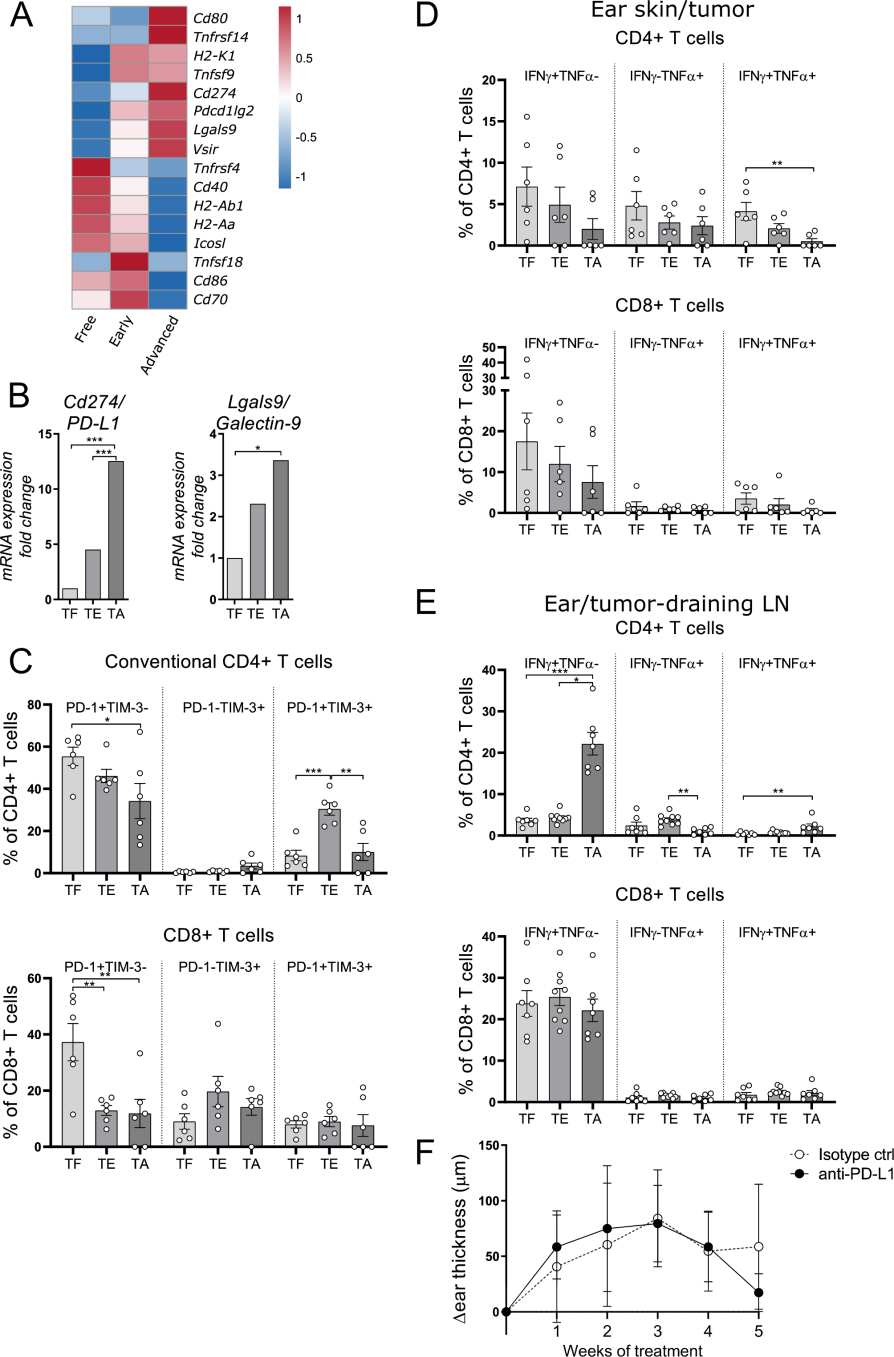
Immunosuppressive molecules increase with tumor progression. (A) RNA-seq analysis was performed with ear skin/tumor tissue from TF (tumor-free), tumor-early (TE) and TA (tumor-advanced) tg(Grm1)EPv mice. The heatmap depicts normalized and relative expression (z-score) levels of several checkpoint ligands. Mean expression for three mice per group is shown. (B) The mRNA levels of programmed death ligand-1 (PD-L1) and Galectin-9 from ear skin/tumors of TF, TE, TA mice were quantified by real-time quantitative PCR. Fold change in comparison to the TF stage is shown for five to six mice per group from one to two independent experiments. (C) Percentages of PD-1+ and TIM-3+ conventional CD4+ and CD8+ T cells from ear skin/tumors of TF, TE, TA tg(Grm1)EPv mice were determined by flow cytometry. Summary graph for seven mice per group from four independent experiments is shown. (D, E) Cell suspensions from ear skin/tumors (D) and draining lymph nodes (LNs) (E) were restimulated in vitro with anti-CD3/anti-CD28 mAbs. The percentages of interferon γ (IFNγ) and tumor necrosis factor α (TNFα) producing CD4+ and CD8+ T cells were analyzed by flow cytometry. Results are from three independent experiments with six to eight mice per group. (F) Tg(Grm1)EPv mice at the transition from TE to TA stage (6.5–7 months old) were treated intraperitoneally with anti-PD-L1 mAb twice per week. Tumor growth was determined by measuring ear thickness changes over time. Results for six mice per group from two independent experiments are shown. Statistical significance was determined using one-way analysis of variance or Kruskal-Wallis analysis (B–E) and two-tailed unpaired Student’s t-test (F). Graphs show the mean ± SE. *p<0.05; **p<0.01; ***p<0.001.

Our earlier work demonstrated that late stage tumors of the tg(Grm1)EPv mice were infiltrated by MDSCs and that these could suppress melanoma-specific T cell responses.^21^ In this study, we aimed at identifying mechanisms of immune evasion that occur earlier in tumor development. For this, we performed RNA-seq of ear skin from tumor-free (TF), tumor-early (TE) and tumor-advanced (TA) tg(Grm1)EPv mice (see online supplemental figure S1A for the different stages). Our RNA-seq analysis of progressing tumors revealed an upregulation of immunosuppressive molecules that can inhibit T cell function (figure 1A). By RT-qPCR we confirmed that the mRNA levels for PD-L1 and Galectin-9, the ligands for the inhibitory molecules PD-1 and TIM-3 respectively, were strongly upregulated in advanced tumors (figure 1B). By flow cytometry we detected PD-L1 expression on multiple cell types, including DCs, CD4+ and CD8+ T cells (online supplemental figure S1B, C). Viable melanoma cells cannot be retrieved after enzymatic digestion in this mouse model, therefore we could not determine the expression of PD-L1 and/or galectin-9 on the surface of tumor cells. Whereas the percentages of tumor-infiltrating CD4+ and CD8+ T cells only slightly changed with tumor progression (online supplemental figure S1D, E), most of the T cells expressed either one of the inhibitory receptors PD-1 and TIM-3 or both (figure 1C). The few regulatory T cells (Tregs) in the tumors mainly expressed TIM-3 (online supplemental figure S1F, G).

To examine whether T cells become functionally impaired with tumor progression, we evaluated their cytokine production after in vitro restimulation with antibodies against CD3 and CD28. A gradual decrease in the percentages of IFNγ and TNFα producing T cells was observed; however, this decrease was statistically significant only for the fraction of CD4+ T cells producing both IFNγ and TNFα and not for the rest of the populations (figure 1D). Furthermore, T cells from tumor-draining LNs produced IFNγ and TNFα even during tumor progression (figure 1E). These results suggest that tumor-infiltrating T cells (TILs) are able to produce cytokines; however, they become functionally impaired due to the presence of immunosuppressive molecules, such as PD-L1. Thus, we treated tg(Grm1)EPv mice at the transition between the TE and the TA stages with a mAb against PD-L1 but this treatment was not sufficient to delay tumor growth (figure 1F). In agreement with a previous study,^7^ this therapy also failed to induce infiltration of CD4+ and CD8+ T cells into the tumor (online supplemental figure S1H).

We conclude that the tg(Grm1)EPv melanoma mouse model does not respond to monotherapy with PD-L1 blockade and that additional mechanisms limit the antitumor immune response in these tumors.

### DCs gradually decrease in melanoma lesions and can be restored by a DC boost approach

**Figure 2:**
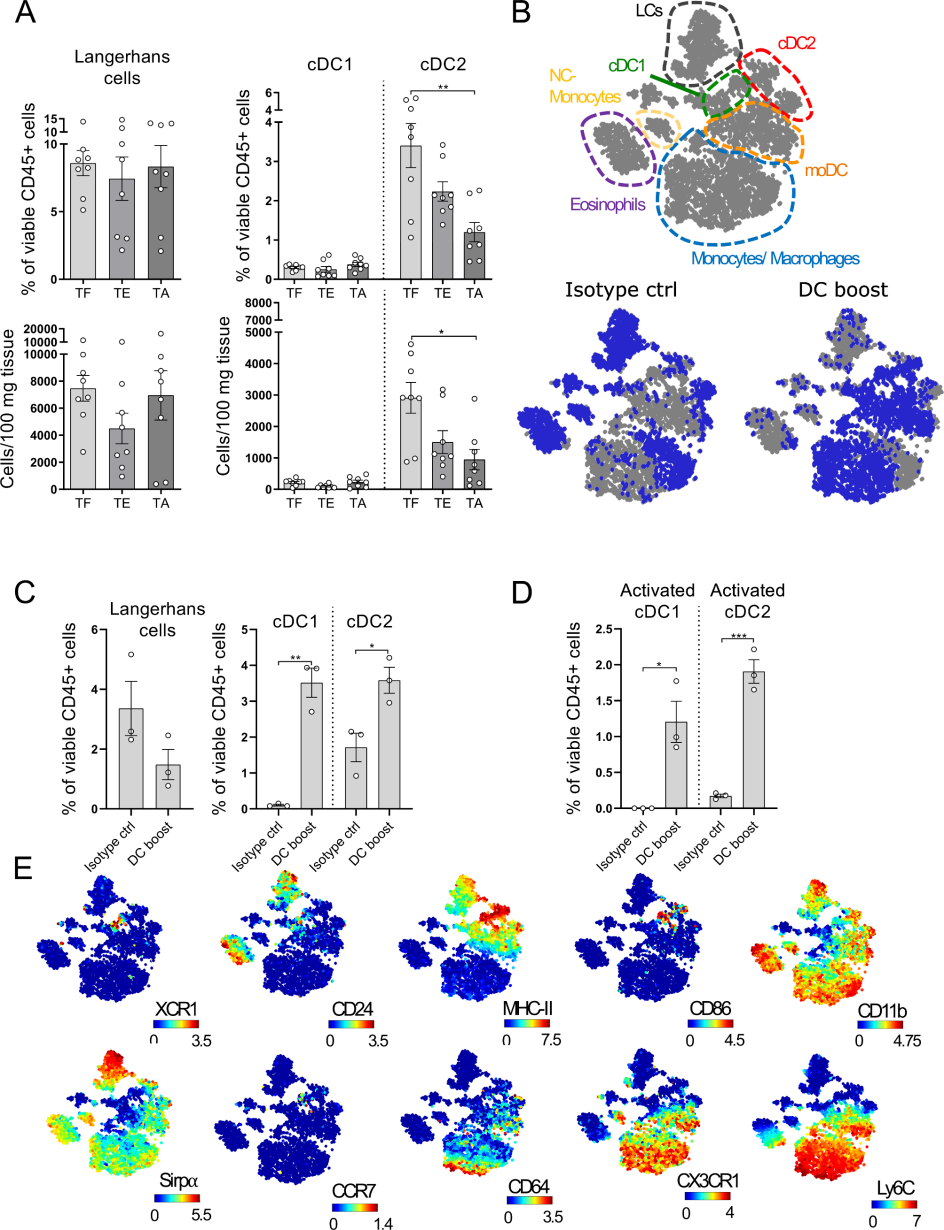
Dendritic cells (DCs) gradually decrease in melanoma lesions and can be restored by a DC boost approach. (A) Ear skin/tumors of TF (tumor-free), tumor-early (TE) and tumor-advanced (TA) tg(Grm1)EPv mice were analyzed by flow cytometry for the percentages of skin DC subsets (gating strategy shown in online supplemental figure S2A). Results from six to eight to 8 mice per group from five independent experiments are shown. (B) tg(Grm1)EPv mice at the transition from TE to TA stage (6.5–7 months old) were treated as illustrated in online supplemental figure S2C. Changes in the myeloid cells were determined by mass cytometry. Top panel: the different populations shown in the viSNE map were identified by manual gating (online supplemental figure S2D). Bottom panels: In blue, the distribution of the identified cells for isotype control (left) and DC boost (right) treated mice are shown. (C) The frequencies of the different skin DC subsets in isotype and DC boost treated mice were determined. (D) The frequencies of activated cDC1 and cDC2 as determined by CD86 expression are shown. (E) Surface expression of XCR1, CD24, MHC-II, CD86, CD11b, Sirpα, CCR7, CD64, CX3CR1 and Ly6C by the various myeloid subsets are shown on the viSNE plots. For (B)–(E) results for three mice from two independent measurements are shown. Statistical significance was determined using one-way analysis of variance or Kruskal-Wallis analysis (A) and two-tailed unpaired Student’s t-test (C and D). Graphs show the mean ± SE. *p<0.05; **p<0.01; ***p<0.001.

A significant decrease in intratumoral DCs has been observed in primary human melanoma lesions and this has been associated with a worse prognosis.^7 10 38^ To examine whether this holds true for the tg(Grm1)EPv mice, we analyzed the changes in the percentages and numbers of the different skin DC subsets during tumor progression. The cDC2 population showed a gradual decrease with tumor growth, whereas LC and cDC1 numbers remained unchanged (figure 2A, gating strategy shown in online supplemental figure S2A). As DCs are crucial in the induction of antitumor immune responses, we investigated whether we could overcome this DC loss. The decrease in dermal cDC2 was pronounced at the transition between the TE and the TA stage (6.5–7 months old), so we examined whether DC numbers in the tumor microenvironment (TME) could be restored during that period. Indeed, systemic administration of Flt3L for 1 week increased the frequencies of dermal cDC2 in the tumor (online supplemental figure S2B). To ensure proper maturation of the recruited DCs, we performed intratumoral injections with the toll-like receptor (TLR)-3 ligand polyI:C and a mAb against CD40 (the treatment with Flt3L and polyI:C/anti-CD40 will be referred to as DC boost therapy from now on, treatment scheme shown in online supplemental figure S2C). We performed CyTOF analysis of the tumors to accurately identify the different myeloid populations.^39^ Samples from both the DC boost and the isotype group were concatenated and a viSNE analysis was performed. Through manual gating (gating strategy shown in online supplemental figure S2D) the major myeloid populations were identified and overlayed on the viSNE map shown in figure 2B. The viSNE plots highlight in blue the most dominant myeloid cell types present in control and DC boost treated mice (figure 2B). The analysis revealed higher percentages of cDC1s, cDC2s and monocytes by the DC boost regimen, whereas LCs remained mostly unchanged (figure 2C and online supplemental figure S2E). These cDC1s and cDC2s had increased expression of CD86, MHC-II and CCR7, suggesting that these cells were highly activated and developed migratory potential towards the tumor-draining LN (figure 2D, E).

10.1136/jitc-2020-000832.supp5 Supplementary data

Thus, in addition to the upregulation of immunosuppressive molecules within the TME, the loss of dermal cDC2 points at impaired antitumor immunity. A DC boost therapy with Flt3L plus polyI:C/anti-CD40 restores the pool of intratumoral DCs with an activated phenotype.

### Boosting DC numbers and function facilitates responsiveness to checkpoint blockade

**Figure 3:**
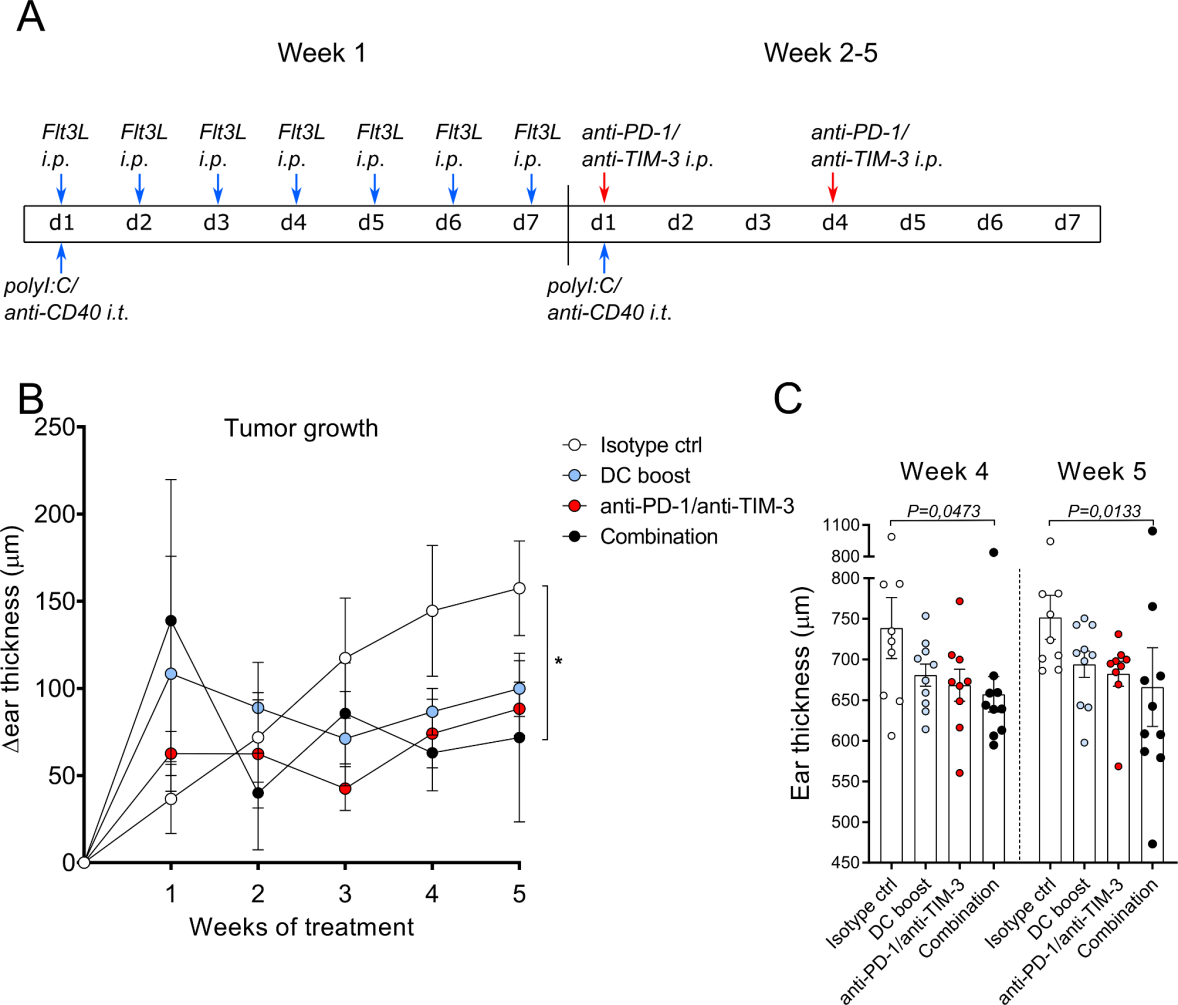
Boosting dendritic cell (DC) numbers and function facilitates responsiveness to checkpoint blockade. (A) The treatment scheme for isotype control, DC boost, checkpoint blockade and combination therapies over 5 weeks is depicted. DC boost consisted of daily injections of 10 µg Fms-related tyrosine 3 ligand (Flt3L) intraperitoneally (i.p.) during the first week of treatment and weekly intratumoral injections of polyI:C and anti-CD40 (25 µg each per mouse). Checkpoint blockade consisted of i.p. injections of 100 µg/mouse of both anti-PD-1 and anti-TIM-3 blocking mAbs and was administered twice per week starting from the second week. Blue arrows indicate DC boost interventions and red arrows indicate checkpoint blockade. Mice in the combination group received treatment with DC boost and checkpoint blockade. Isotype control therapy consisted of PBS instead of Flt3L and polyI:C and isotype control antibodies for anti-CD40, anti-PD-1/anti-TIM-3. (B) Tg(Grm1)EPv mice at the transition from tumor-early (TE) to tumor-advanced (TA) stage were treated and ear thickness changes for 8–10 mice per group from two independent experiments were measured weekly. *p<0.05. (C) Ear thickness measured at weeks 4 and 5 is shown. Statistical significance was determined using the Kruskal-Wallis analysis for (B) and (C). PD-1, programmed cell death protein-1; TIM-3, T-cell immunoglobulin and mucin-domain containing-3.

Our data so far identified two potential mechanisms that could hamper antitumor immune responses in the tg(Grm1)EPv mouse model: the upregulation of molecules that can inhibit T cell function in the tumor and the decrease of intratumoral DCs. As monotherapy with anti-PD-L1 mAb was not sufficient to delay tumor growth, we sought to boost DC function and at the same time block inhibitory molecules on T cells. For this, we treated tg(Grm1)EPv mice with a DC boost therapy consisting of Flt3L daily for 1 week and weekly intratumoral injections with polyI:C/anti-CD40 for 5 weeks. As intratumoral T cells of the tg(Grm1)EPv mice expressed both PD-1 and TIM-3 (figure 1C), we also tested checkpoint blockade therapy by administering blocking mAbs for both molecules twice a week for weeks 2–5. An additional group that received both treatments (combination) was also included (treatment scheme in figure 3A). Although DC boost and checkpoint blockade single therapies delayed tumor growth, only the combination treatment inhibited tumor growth significantly in comparison to the isotype control group (figure 3B, C).

These results indicate that the combination of checkpoint blockade with an approach that restores the pool of activated DC can control tumor growth.

### The DC boost results in increased infiltration of activated DCs and T cells into tumors

**Figure 4:**
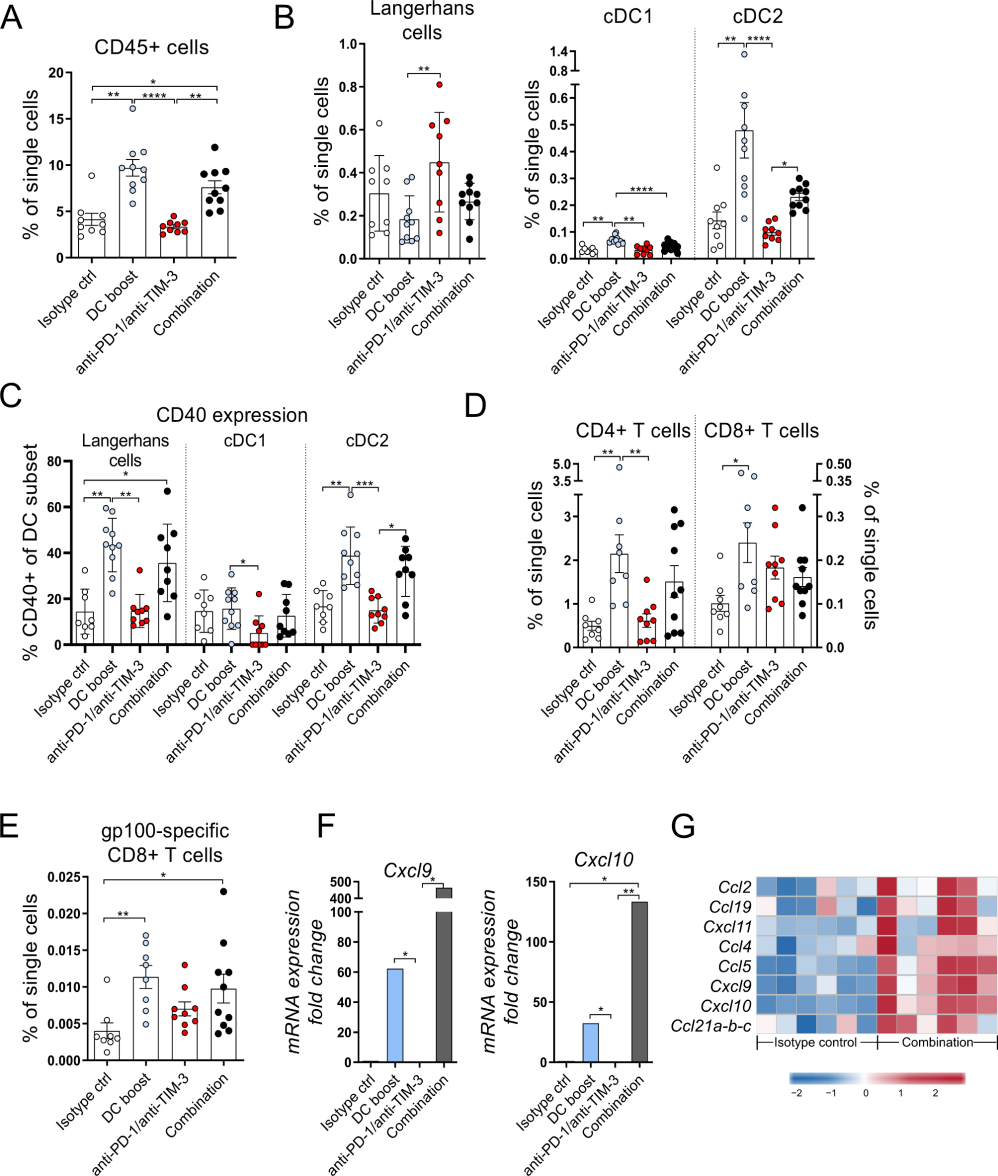
The DC boost results in increased infiltration of activated dendritic cells (DCs) and T cells into tumors. Tg(Grm1)EPv mice at the transition from tumor-early (TE) to tumor-advanced (TA) stage were treated for 5 weeks as described in figure 3A. (A–E) Frequencies of the CD45+ immune cells (A), skin DC subsets (B), CD40 expression on DC subsets (C), CD4+ and CD8+ tumor-infiltrating T cells (D) and gp100-specific CD8+ T cells (E) were determined by flow cytometry. For (A)–(E), n=8–10 mice per group from two independent experiments. (F) The mRNA levels for *Cxcl9* and *Cxcl10* were quantified by real-time quantitative PCR. Fold change in comparison to the isotype control is shown for n=4–6 mice per group from three independent experiments. (G) Tumor RNA from isotype control and combination therapy treated mice was analyzed by microarray. Heatmap from microarray data displaying the normalized and relative expression (z-score) of genes associated with lymphocyte trafficking. n=6 mice per group from two independent experiments. For (A)–(F), statistical significance was determined using one-way analysis of variance or Kruskal-Wallis analysis. Graphs show the mean ± SE. *p<0.05; **p<0.01; ***p<0.001; ****p<0.0001. PD-1, programmed cell death protein-1; TIM-3, T-cell immunoglobulin and mucin-domain containing-3.

To understand the immunological processes mediating the delay in tumor growth in the tg(Grm1)EPv mouse model, we examined the changes in the immune cell composition of the tumors after 5 weeks of treatment. First of all, the DC boost approach led to a statistically significant increase of the total CD45+ immune cells that was persistent in the combination group (figure 4A). When we looked into detail, we detected more intratumoral cDC1 and cDC2 (figure 4B) that expressed high levels of CD40 (figure 4C). Apart from the increased frequencies of DCs, we observed a recruitment of CD4+ and CD8+ T cells (figure 4D) as well as CD4+ Tregs and MDSCs in the tumors (online supplemental figure S3A, B). Nevertheless, the delay in tumor growth by the combination treatment (figure 3B, C) suggests that antitumor immunity overrules immunosuppression. Indeed, the increased ratio of CD4+ T cells/Tregs in the same groups indicates a dominance of T helper cells over regulatory, immunosuppressive cells (online supplemental figure S3C). This was not reflected in the ratio of CD8+ T cells/Tregs (online supplemental figure S3D). Along with the intratumoral CD8+ T cells, we detected increased frequencies of gp100-specific CD8+ T cells in the tumors of mice that received the DC boost treatment, suggesting the induction of a tumor-specific immune response (figure 4E). Previous studies have shown that infiltrating DCs produce chemokines crucial for the recruitment of T cells.^6 40^ RT-qPCR analysis for *Cxcl9* and *Cxcl10* showed that the DC boost approach upregulated both chemokines and this was even more pronounced in the combination treatment group (figure 4F). We observed a similar trend for the protein levels of these chemokines in the tumor tissue (online supplemental figure S3E). Microarray analysis of the tumors for additional chemokines required for T cell recruitment into tissues showed that their expression was also increased in the combination treatment group (figure 4G).

10.1136/jitc-2020-000832.supp6 Supplementary data

Altogether, our data outline that the DC boost approach is essential to recruit activated DCs and T cells to the tumors to mediate anti-tumor immunity.

### DC boost treatment improves antitumor immune responses in the draining lymph node

**Figure 5:**
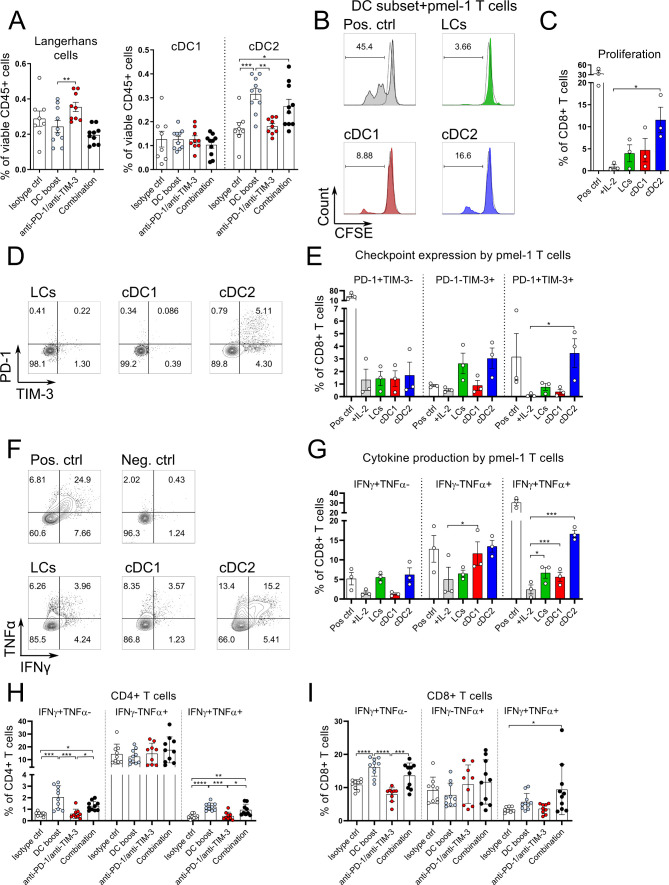
DC boost treatment improves antitumor immune responses in the draining lymph node. (A) Tg(Grm1)EPv mice at the transition from tumor-early (TE) to tumor-advanced (TA) stage were treated for 5 weeks as described in figure 3A. Percentages of migratory skin dendritic cell (DC) subsets were determined by flow cytometry in the tumor draining lymph nodes (LNs). (B–G) Tg(Grm1)EPv/Langerin-EGFP mice at the transition from TE to TA stage were treated with the DC boost regimen as in online supplemental figure S2C. The three main migratory skin DC subsets (LCs, cDC1, cDC2) were sorted from the tumor-draining LNs (see online supplemental figure S4C for sorting strategy). Sorted DCs were cocultured with gp100-specific CD8+ T cells isolated from pmel-1 mice in a ratio of DCs:T cells 1:3 for 3 days. As a negative control, T cells were cultured without stimulation but with IL-2 only (+IL-2). T cells cocultured with gp100 peptide loaded DCs served as positive control (Pos ctrl). (B) Representative histograms showing carboxyfluorescein succinimidyl ester (CFSE) dilution of CD8+ T cells in response to the different DC subsets. (C) Percentages of proliferated CD8+ T cells from three independent experiments. (D) Representative dot plots showing the expression of PD-1 and TIM-3 on the CD8+ T cells after 3 days of coculture with the different DC subsets. (E) Percentages of PD-1+ and TIM-3+ CD8+ T cells from three experiments. (F) At day 3 of coculture, CD8+ T cells were restimulated with anti-CD3/anti-CD28 mAbs and production of IFNγ and TNFα was measured by intracellular flow cytometry. Representative dot plots showing cytokine production by T cells in response to the different DC subsets. (G) Percentages of IFNγ+ and of TNFα+ CD8+ T cells from three experiments are shown. (H and I) LN cell suspensions of Tg(Grm1)EPv mice at the transition from TE to TA stage that were treated for 5 weeks as described in figure 3A were restimulated in vitro with anti-CD3/anti-CD28 mAbs. Percentages of IFNγ+ and TNFα+ CD4+ T cells (H) and CD8+ T cells (I) were analyzed by flow cytometry. Statistical significance was determined using one-way analysis of variance (ANOVA) or Kruskal-Wallis analysis (A, H, I), Friedman test (C and E) and repeated-measures one-way ANOVA (G). Graphs show the mean ± SE. *p<0.05; **p<0.01; ***p<0.001; ****p<0.0001. IFNγ, interferon γ; PD-1, programmed cell death protein-1; TIM-3, T-cell immunoglobulin and mucin-domain containing-3; TNFα, tumor necrosis factor α.

Our results so far show that the DC boost approach modulates the immune infiltrate in tumors in favor of activated DC and T cells. Moreover, Gene Set Enrichment Analysis (GSEA) performed on the microarray data showed that tumors treated with the combination approach, were enriched for pathways related to antigen processing and (cross-) presentation (online supplemental figure S4A, B). As the tumor-draining LN is the location where de novo T cell responses are initiated, we next investigated the immunological changes there. We found that after 5 weeks of treatment the migratory cDC2 increased in frequencies whereas the cDC1 subset and LCs were unchanged (figure 5A, gating strategy in online supplemental figure S4C). We observed a similar pattern at earlier time points following the DC boost approach (online supplemental figure S4D, E). Previous studies reported that within tumors, the main DC subset involved in the cross-presentation of tumor antigens is the cDC1.^6 7^ However, the priming ability of the various migratory skin DCs in the tumor-draining LNs is still incompletely understood, so we aimed to investigate the cross-priming abilities of the different migratory DC subsets in the tumor-draining LNs after DC boost treatment. Tg(Grm1)EPv/langerin-EGFP mice that allow cell sorting of Langerin+ DC/LC were used at the transition from TE to TA and treated with the DC boost regimen for 1 week (see scheme in online supplemental figure S2C). One day after the end of the treatment, we sorted the three main migratory DC subsets from the tumor-draining LNs (see online supplemental figure S4C for sorting strategy) and tested them for their ability to cross-present tumor-derived antigen to gp100-specific TCR-transgenic CD8+ T cells.^23^ The in vitro T cell proliferation confirmed that cDC1 cross-present tumor antigens and induce proliferation of gp100-specific CD8+ T cells. In addition, we observed that the DC boost approach reinforced migratory skin cDC2 to perform this task (figure 5B, C). Furthermore, the cDC2 induced coexpression of PD-1 and TIM-3 on CD8+ T cells, making them susceptible to checkpoint blockade therapy (figure 5D, E). When we restimulated CD8+ T cells from these cocultures with anti-CD3/anti-CD28, we found that IFNγ and TNFα production was induced by all three migratory DC subsets, with migratory cDC1 and cDC2 being the most potent in the induction of the double-producers (figure 5F, G). By examining the functional properties of T cells in the tumor-draining LNs in mice that had received treatments for 5 weeks, we detected higher numbers of IFNγ and TNFα-producing CD4+ and CD8+ T cells after in vitro restimulation (figure 5H, I).

These observations reveal that even after 5 weeks of treatment, there is continuous migration of dermal DCs to the tumor-draining LNs, with this effect being more pronounced for the cDC2 subset. The DC boost approach enables all skin DC subsets to cross-present endogenous gp100 tumor-associated antigen to CD8+ T cells leading to their activation. Furthermore, it enhances the in vivo function of CD4+ and CD8+ T cells in the tumor-draining LN, an important location for T cell priming.

### Combination of DC boost with checkpoint blockade of PD-1 and TIM-3 results in a higher cytotoxic activity in the tumor

**Figure 6:**
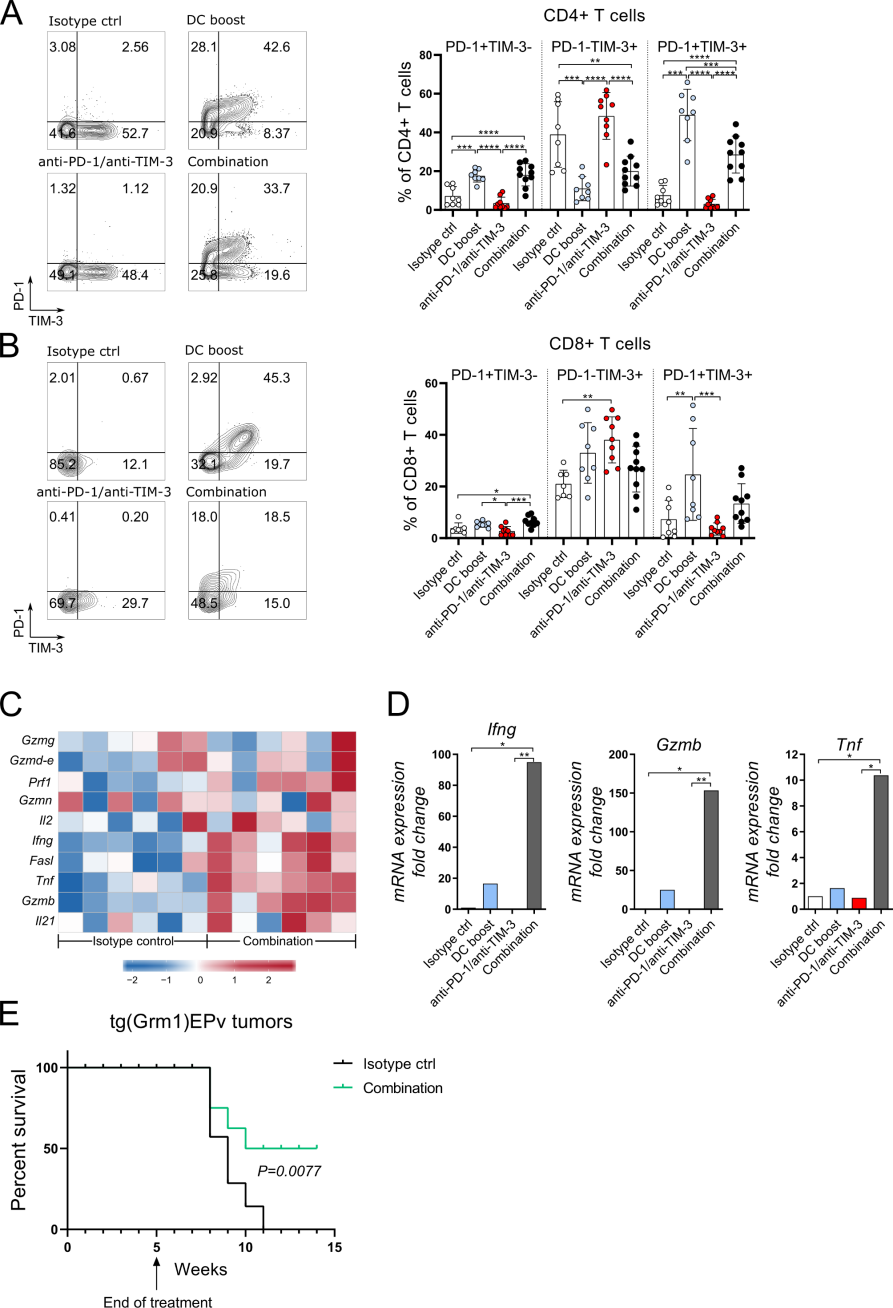
Combination of DC boost with checkpoint blockade of programmed cell death protein-1 (PD-1) and T-cell immunoglobulin and mucin-domain containing-3 (TIM-3) results in a higher cytotoxic activity in the tumor. Tg(Grm1)EPv mice at the transition from tumor-early (TE) to tumor-advanced (TA) stage were treated for 5 weeks as described in figure 3A. Representative contour plots and summary graphs depicting PD-1 and TIM-3 expression of CD4+ (A) and CD8+ (B) T cells from tumors of mice from different treatment groups. n=8–10 mice per group from two independent experiments. (C) Tumor RNA from isotype control and combination therapy treated mice was analyzed by microarray. The heatmap depicts the normalized and relative expression (z-score) of genes associated with immune-mediated cytotoxicity for six mice per group from two experiments. (D) The mRNA levels for *Ifng*, *Tnf* and *GzmB* were quantified by real-time quantitative PCR. Fold change in comparison to the isotype control is shown for four to six mice per group from three independent experiments. (E) Long-term survival of tg(Grm1)EPv mice after cessation of treatment at week five in isotype control and combination treatment groups (treatment scheme in figure 3A). Six mice per group from one experiment are shown. Statistical significance was determined with one-way analysis of variance or Kruskal-Wallis analysis (A, B and D) or log-rank (Mantel-Cox) test (E). Graphs in (A), (B) and (D) show the mean±SE. *p<0.05; **p<0.01; ***p<0.001; ****p<0.0001.

To understand better the immunological processes in the treated tumors, we analyzed the tumor-infiltrating CD4+ and CD8+ T cells. Both subsets contained more PD-1 and TIM-3 positive cells when compared with the isotype control group, an effect that was most significant in the DC boost and combination groups (figure 6A, B). Thus, the DC boost approach induced these surface molecules, and especially of PD-1, which in turn rendered T cells more responsive to checkpoint blockade. A detailed analysis of genes important for T cell function was performed with microarray and RT-qPCR. Several genes related to immune cell-related cytotoxicity, for example, *Gzmb*, *Fasl*, *Prf1* and activation of T cells, for example, *Il2*, *Ifng*, *Tnf*, were upregulated in tumors that received the combination therapy (figure 6C). RT-qPCR analysis revealed that in the DC boost group, the mRNA expression of *Ifn, Gzmb* and *Tnf* was increased (figure 6D). But in fact, the combination therapy with checkpoint blockade therapy was essential to achieve maximal expression of these molecules (figure 6D). To investigate if the T cells are able to mediate antitumor immunity, we injected transplantable B16.OVA melanoma cells at week 4 of treatment into the flank skin of tg(Grm1)EPv mice and terminated treatment at week five according to the original treatment scheme (figure 3A). Growth of B16 tumors was delayed in non-treated tg(Grm1)EPv mice due to a pre-existing antitumor immune response when compared with naive C57BL/6 mice (online supplemental figure S5A). Still, B16 tumors eventually grew and prolonged survival could be achieved only in mice that received either the DC boost or the combination treatment (online supplemental figure S5B). Finally, we wanted to translate this into the transgenic melanoma model and investigated the survival of tg(Grm1)EPv with spontaneous tumors after therapy cessation and we observed that the combination treatment prolonged survival (figure 6E).

10.1136/jitc-2020-000832.supp7 Supplementary data

These results demonstrate that the DC boost approach drives alterations in the TME that increase the immunogenicity of tumors and renders them more responsive to immunotherapy with checkpoint inhibitors. On addition of anti-PD-1 and anti-TIM-3 to the DC boost treatment, the cytotoxic capacity of the T cells is released leading to efficient tumor growth control.

## Discussion

The current research focus in clinical tumor immunology is mainly centered on TILs and how their function can be restored with checkpoint blockade.^2 3^ Recently, the importance of DCs for this type of immunotherapy was revealed,^5^ and several reports demonstrated a dominant role for cDC1 in the cross-presentation of tumor antigens in the tumor microenvironment (TME).^6–8^ Nevertheless, the role of the migratory skin DC in priming melanoma-specific T cell responses in the tumor-draining LNs is just partly understood. In our study, we used the tg(Grm1)EPv spontaneous melanoma mouse model which allows for the in-depth assessment of coevolution of the tumor-immune microenvironment, due to slow and gradual melanoma growth.^19 21^ Characterization of the TME demonstrated an upregulation of T cell inhibitory molecules during tumor progression with a concomitant loss of cDC2 that might facilitate tumor immune evasion. Indeed, we report here that boosting the numbers and activation of intratumoral cDC1 and cDC2 is key for the success of immunotherapy with checkpoint blockade. Moreover, the DC boost approach equips not only the migratory cDC1 but also the migratory cDC2 in the tumor-draining LNs of the tg(Grm1)EPv mouse model with the ability to prime gp100-specific CD8+ T cells. In the end, we demonstrate that a combination therapy with DC boosting reagents and checkpoint blockade antibodies restored T cell responses, delayed tumor growth and prolonged survival of tumor-bearing mice.

We have previously shown that MDSCs are recruited to advanced melanoma lesions of the tg(Grm1)EPv mice and that these MDSCs are able to inhibit gp100-specific CD8+ T cell responses.^21^ In the present study, we examined how antitumor immune responses are impaired at the interface between early and advanced tumor stages. One major mechanism identified was the upregulation of PD-L1 and galectin-9 in the tumor. As monotherapy with anti-PD-L1 was not sufficient to control tumor growth, we examined further changes that may occur in the tumor-immune microenvironment. A decrease in skin DCs has already been described in earlier studies that characterized the immune infiltrate in primary human melanoma lesions.^7 10^ In line with this, tumor progression in the tg(Grm1)EPv mice is accompanied by a continuous loss of dermal cDC2, the most abundant DCs within the growing melanoma lesions. As DCs are key mediators of antitumor immune responses,^6–8 10^ we sought to increase their numbers and activation. For this, we used treatment with Flt3L which has been described to expand all DC subsets except LCs in lymphoid organs and peripheral tissues, including tumors.^7 18^ Furthermore, we used an adjuvant mix consisting of polyI:C and a mAb against CD40, as previous studies reported that activating DCs in tumors is essential for efficient and long-lasting immune responses.^41^ We report here that this DC boost approach (Flt3L plus polyI:C/anti-CD40) enhanced the numbers and activation of intratumoral dermal cDC1 and cDC2.

A necessary step in the induction of antitumor immune responses is the priming of T cells in the tumor-draining LN. The capacity of migratory cDC1 and cDC2 to cross-present antigen has been observed before in the steady state.^42^ However, it is not entirely understood what exactly is the role of the migratory skin DC subsets in the priming of melanoma-specific T cells and how this role changes in response to immunotherapy. In our study, we observed higher frequencies of migratory cDC2 in the tumor-draining LNs after DC boost treatment. Through co-cultures of sorted migratory skin DC subsets with gp100-specific CD8+ T cells after DC boost treatment, we observed that both migratory dermal DC, cDC1 and cDC2, and to a lesser extent LCs, can cross-present gp100 tumor-associated antigen. Thus, the DC boost approach can enhance the cross-presenting capacity of all skin DC subsets in the tumor-draining LN, an essential step in the priming process of antitumor immunity.

In line with this, the DC boost approach is able to partially control tumor growth in the tg(Grm1)EPv mouse model. More specifically, we observed recruitment of CD4+ and CD8+ T cells into treated tumors that expressed to a high extent PD-1 and/or TIM-3, rendering them susceptible to subsequent checkpoint blockade therapy. Thus, we designed a combination therapy consisting of the DC boost and checkpoint blockade antibodies against PD-1 and TIM-3. Indeed, this combination treatment significantly delayed tumor growth. The reason for better efficacy was the strong induction of cytokines and cytotoxic molecules, including granzyme B, IFNγ and TNFα in the treated tumors. In line with this, mice treated with combination therapy were partly protected against a subsequent challenge with transplantable B16.OVA melanoma cells indicating the induction of melanoma-specific T cells. Indeed, we detected infiltration of gp100-specific CD8+ T cells into the tg(Grm1)EPv-treated tumors. Moreover, we demonstrated prolonged survival of tg(Grm1)EPV mice even after cessation of therapy suggesting long-term control of spontaneous tumors.

A recent study demonstrated that cDC2 are crucial for the initiation of CD4+ T cell-driven anti-melanoma responses and they correlate with a better response to anti-PD-1 therapy; Treg abundance in steady state tumors is a factor limiting the ability of cDC2 to initiate CD4+ T cell responses.^43^ Our results show that the DC boost approach enhances not only the numbers and activation of intratumoral DC and T cells but also the frequencies of intratumoral Tregs. Our results however indicate a dominance of conventional CD4+ T cells over CD4+ Tregs. Along with the increase in Tregs, we observed an increase in the frequencies of MDSCs. Previous studies have shown that treatment with polyI:C and/or anti-CD40 can limit the suppressive capacity of these cells.^44 45^ It is therefore likely that the DC boost treatment used here limits MDSC-mediated immunosuppression. Nevertheless, future studies should address the exact mechanisms that mediate the protection of conventional CD4+ and CD8+ T cells from immunosuppression mediated by Tregs and MDSCs, as this will provide insight on how specific therapies can limit immunosuppression and enhance responsiveness to checkpoint blockade therapy.

Our study, which employed the tg(Grm1)EPv spontaneous melanoma mouse model, provides strong evidence that enhancing DC numbers and activation can improve the recruitment of intratumoral T cells by upregulation of chemokines required for the recruitment of T cells, including the type I IFN-inducible CXCL9 and 10 chemokines.^46^ As checkpoint blockade alone did not mediate these effects, the DC boost approach appears to be key in this process. Still, for full T cell function in regard to cytokines and cytotoxicity the addition of checkpoint blockade therapy was required. Thus, we conclude that DCs are essential for efficient checkpoint blockade therapy by equipping T cells with cytotoxic properties. Further studies with additional melanoma mouse models where tumor growth is driven by different mutations such as the Braf^V600E^-Pten^-/-^ model,^47^ along with the use of mouse models that allow for the ablation of specific DC subsets should evaluate the potential of the different DC subsets in both tumors and tumor-draining LNs in driving T cell responses during immunotherapy.

## Conclusions

Our data demonstrate that DCs are crucial determinants of tumor immunogenicity; upon DC boost treatment, there is an infiltrate of activated DCs and CD4+ and CD8+ T cells in the tumors, whereas the migratory skin DCs, and especially the dermal cDC1 and cDC2, acquire the ability to prime CD8+ T cell responses in the tumor-draining LN. Therefore, therapies to enhance responsiveness to checkpoint blockade may well benefit from a component that boosts the numbers and function of DCs. Administration of recombinant Flt3L was shown to be effective in the expansion of DCs and safe for healthy volunteers,^48^ increase the frequencies of peritumoral DCs in metastatic colon cancer patients^49^ and restore responsiveness to anti-PD-1 therapy in patients with lymphoma when combined with radiotherapy and administration of polyI:C.^50^ Ongoing clinical trials will reveal whether Flt3L in combination with other immunostimulatory agents including TLR ligands and anti-CD40 can induce durable clinical responses and prolong survival in patients with melanoma.

10.1136/jitc-2020-000832.supp8 Supplementary data
