# Supplementary material for: Skin dendritic cells in melanoma are key for successful checkpoint blockade therapy
Source: J Immunother Cancer. 2021 Jan 6;9(1):e000832. doi: 10.1136/jitc-2020-000832 (PMC7789456; doi:10.1136/jitc-2020-000832)
Supplement: Supplementary data [file jitc-2020-000832supp001.pdf]

Supplementary Figure legends

Supplementary Figure S1. A. Representative pictures of C57BL/6 mice and tg(Grm1)EPv mice at the different tumor stages and H&E stainings from ear skin/tumor sections (x40). **B.** Frequencies of Langerhans cells, cDC1 and cDC2 expressing PD-L1 are shown, N=8 mice per group from three independent experiments. **C.** Frequencies of CD4+ and CD8+ T cells expressing PD-L1 from N=7 mice per group from three independent experiments are shown. **D.** Gating strategy used for the identification of CD4+ and CD8+ T cells in skin/tumor cell suspensions. **E.** Percentages and numbers of CD4+ and CD8+ T cells in the ear skin/tumors of tg(Grm1)EPv mice from 12-13 mice per group from 7 independent experiments is shown. **F.** Representative contour plots showing frequencies of CD4+ Tregs in the ear skin/tumors of the tg(Grm1)EPv mice at different stages. **G**. Frequencies of CD4+ Tregs expressing PD-1 and/or TIM-3 at the different tumor stages. N=6 mice from two independent experiments. **H.** Percentages of tumor-infiltrating CD4+ and CD8+ T cells following treatment with anti-PD-L1 mAb are shown. N=6 mice per group from two independent experiments. Statistical significance was determined using one-way ANOVA or Kruskal-Wallis tests (B, C, E, G, H). Graphs show the mean +/- standard error, *$p < 0.05$, **$p < 0.01$.

Supplementary Figure S2. A. Gating strategy used for the identification of the different skin DC subsets in skin/tumor cell suspensions by flow cytometry. **B.** tg(Grm1)EPv mice at the transition of TE to TA stage received 7 daily i.p. injections of Flt3L. At the end of the treatment the frequencies of Langerhans cells, cDC1 and cDC2 subsets in the tumors were determined by flow cytometry. N=10 mice per group from 2 independent experiments. **C.** Scheme for short-term DC boost treatment: Mice received 7 daily i.p. injections of Flt3L and 2 i.t. injections of polyI:C/anti-CD40 at day 1 and 8. On day 9, tumors and LNs were used for further analysis. **D.** Gating strategy used for the identification of the different myeloid populations with mass cytometry. **E.** Frequencies of monocytes, non-classical monocytes and eosinophils in the control (PBS + isotype mAb) and DC boost-treated mice were determined by mass cytometry; N= 3 mice from two independent

measurements. Statistical significance was determined using two-tailed unpaired student's t-test (B and E), Graphs show the mean +/- standard error, ***p < 0.001.

Supplementary Figure S3. Tg(Grm1)EPv mice at the transition from TE to TA stage were treated for 5 weeks as described in Fig. 3A. Frequencies of **A**. Regulatory CD4+ T cells, N=4-8 mice per group from 3 independent experiments, **B.** Polymorphonuclear MDSC (PMN-MDSC), Monocytic MDSC (M-MDSC), N= 6-7 mice per group from 1 experiment are shown. **C.** and **D.** Ratios of Conventional CD4+ T cells (C) and of CD8+ T cells (D) over CD4+ Tregs are shown, N=4-8 mice per group from 3 independent experiments. **E.** Protein levels for the chemokines CXCL9 and CXCL10 were measured in tumor lysates of tg(Grm1)EPv mice that were treated as in Fig. 3A, N= 6-7 mice per group from 1 experiment. Statistical significance was determined with one-way ANOVA or Kruskal-Wallis analysis, graphs show the mean +/- standard error, *p < 0.05, **p < 0.01.

Supplementary Figure S4. A and B. Tg(Grm1)EPv mice at the transition from TE to TA stage were treated for 5 weeks as described in Fig. 3A and tumor RNA from isotype control and combination therapy treated mice was analyzed by microarray. Gene Set Enrichment Analysis (GSEA) was performed. **A.** Plot shows enrichment of genes related to antigen cross-presentation. P-value = 0.0, FDR value=0.06. **B.** Plot shows enrichment of genes related to antigen processing and presentation via MHC I molecules. P value = 0.004. **C-E.** Migratory skin DC subsets were detected according to their CCR7 expression in tumor draining LNs. **C.** The gating strategy for skin DC subset analysis in the tumor draining LN and for the sorting of the different migratory skin DC subsets is shown. **D.** and **E.** Migratory skin DC subsets were quantified in the tumor draining LN after 1 week (**D**) and 2 weeks of DC boost therapy (**E**). Results for 3-4 mice per group from 1-2 independent experiments. Statistical significance was determined with two-tailed unpaired student's t-test (D, E), graphs show the mean +/- standard error, **p < 0.01.

Supplementary Figure S5. Tg(Grm1)EPv mice were treated for 5 weeks as illustrated in Fig. 3A and at week 4 were injected s.c. with $1.5x10^5$ B16.OVA tumor cells into flank skin. **A.** Survival

curves of isotype-treated tg(Grm1)EPv mice compared to naive, untreated C57BL/6 are shown. **B.** Survival curves of tg(Grm1)EPv mice treated with the DC boost, checkpoint blockade and combination approaches in comparison to isotype control mice are shown. N= 4-5 mice from 1 experiment are shown. Statistical significance was determined with the Log-rank (Mantel-Cox) test, the respective P values are shown.
