# Supplementary figures and images for: Skin dendritic cells in melanoma are key for successful checkpoint blockade therapy
Source: J Immunother Cancer. 2021 Jan 6;9(1):e000832. doi: 10.1136/jitc-2020-000832 (PMC7789456; doi:10.1136/jitc-2020-000832)

## Figure S1

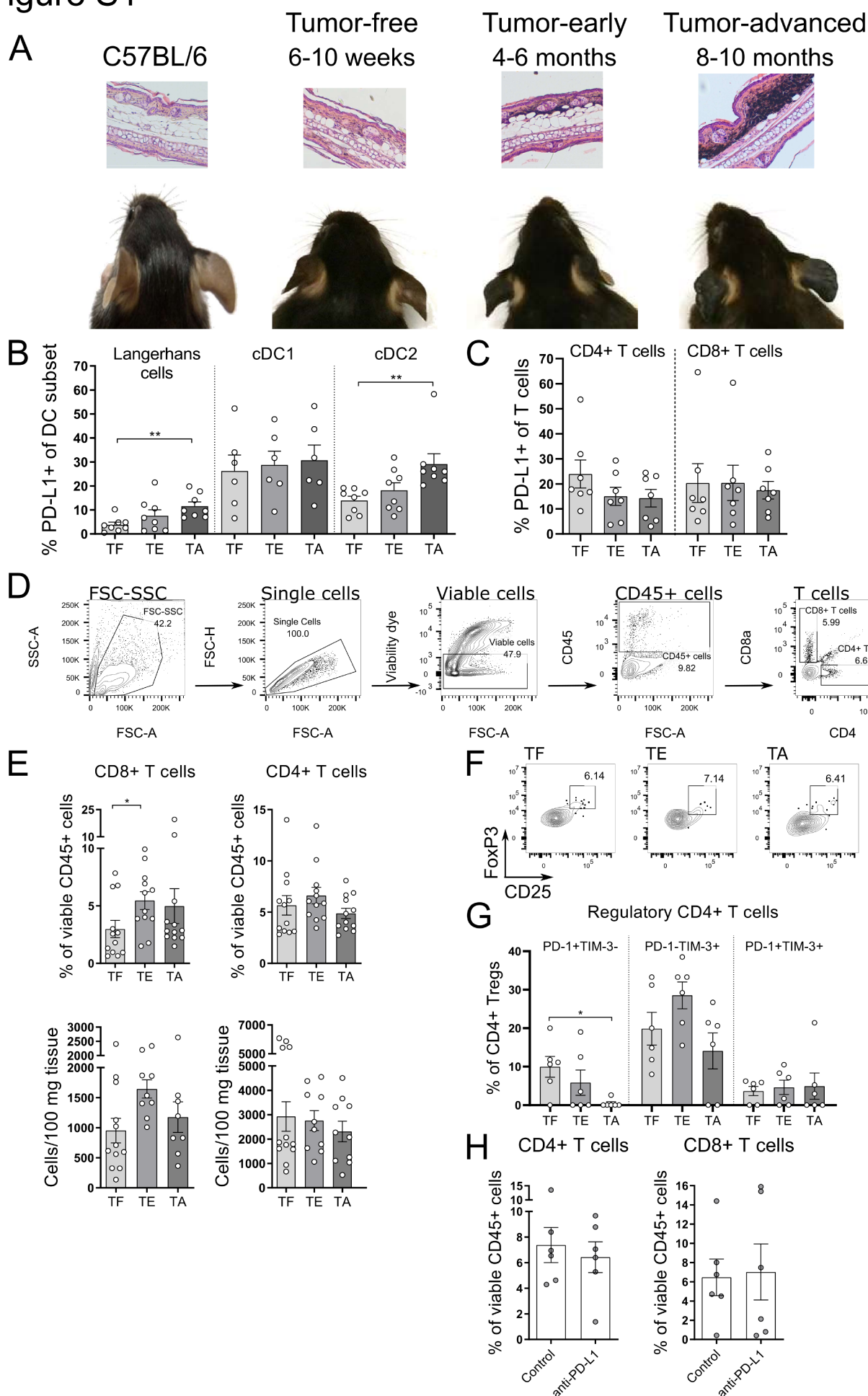

Supplement: Supplementary data [file jitc-2020-000832supp002.pdf]

Figure S4

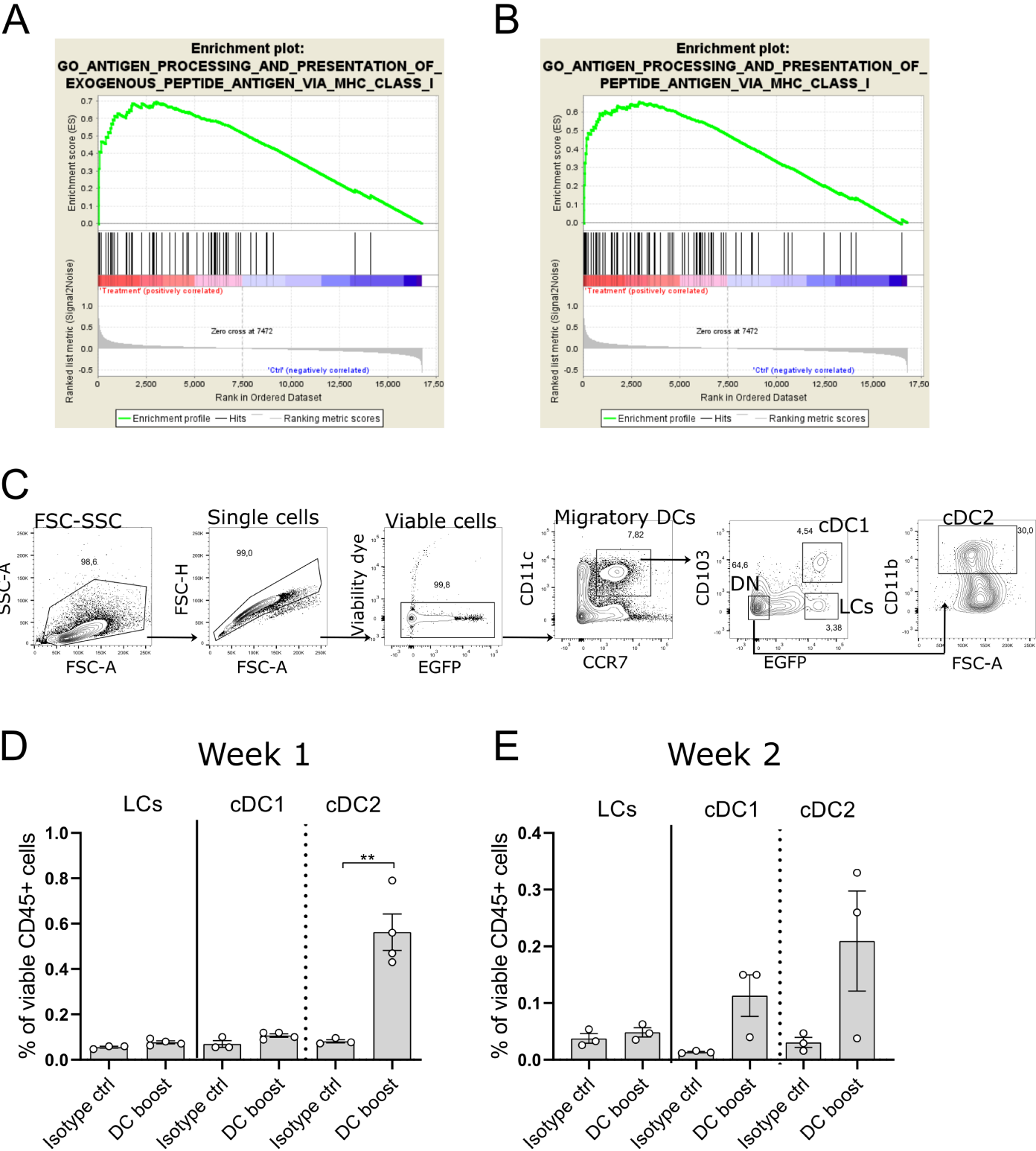

Supplement: Supplementary data [file jitc-2020-000832supp004.pdf]

Figure S2

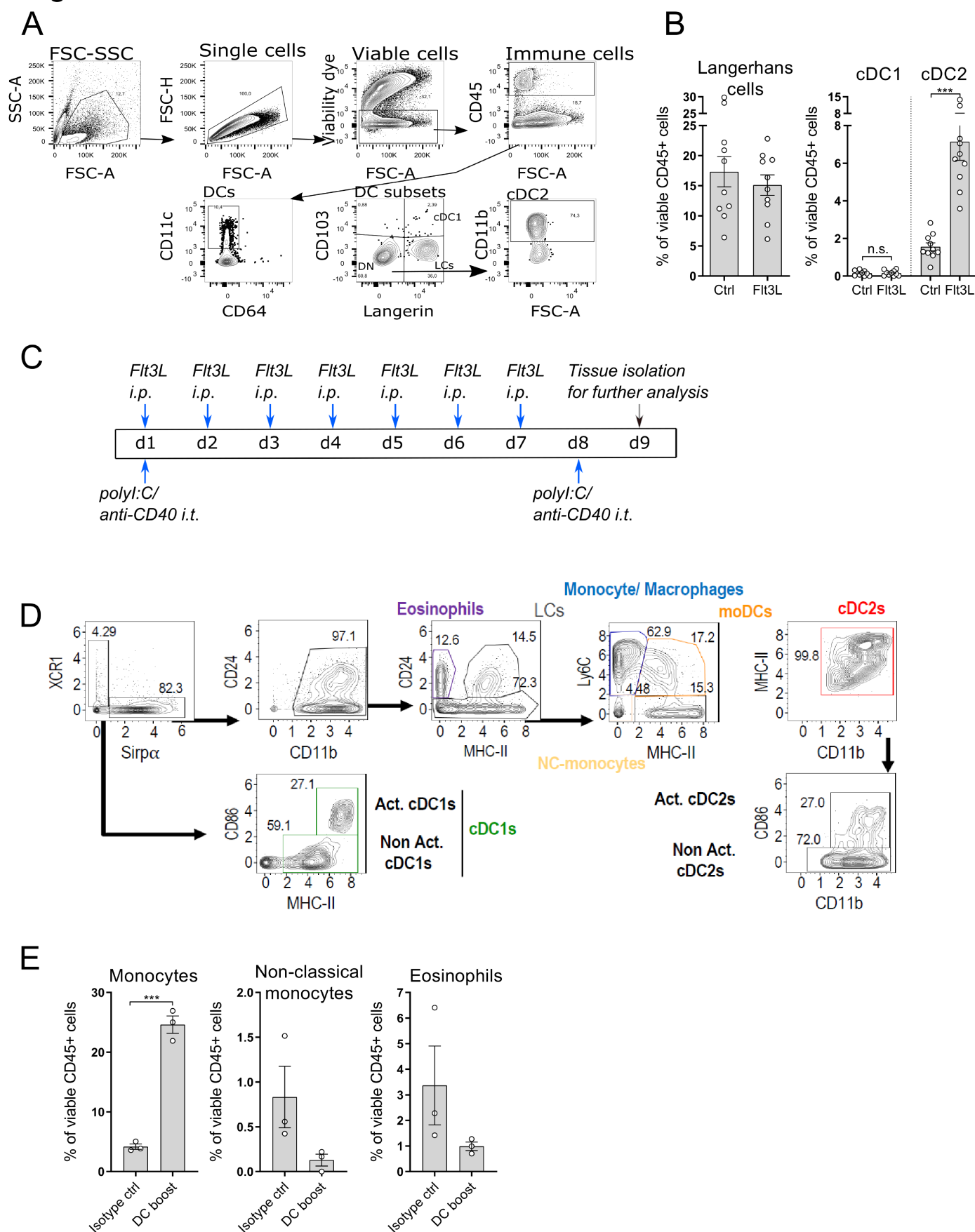

Supplement: Supplementary data [file jitc-2020-000832supp005.pdf]

Figure S3

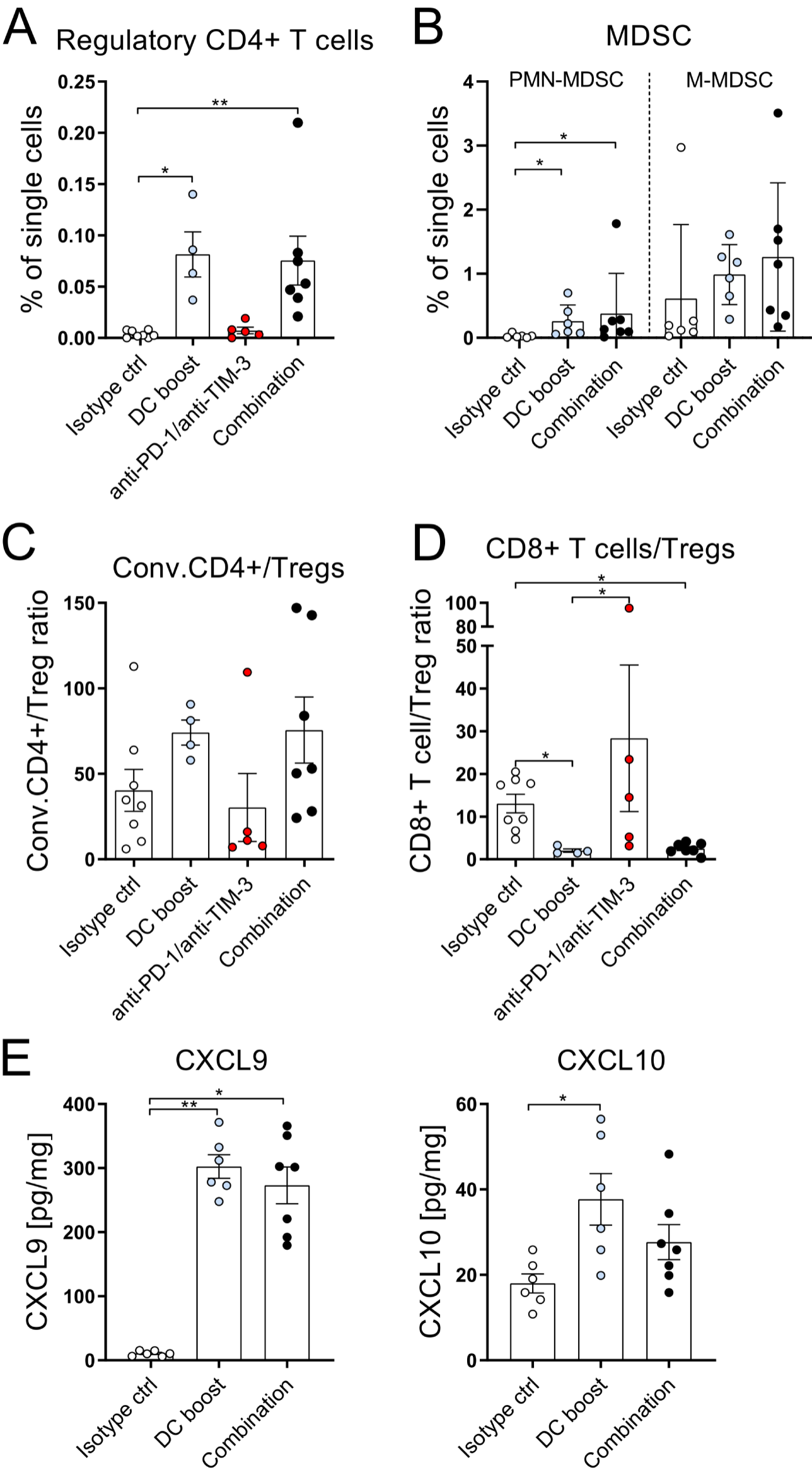

Supplement: Supplementary data [file jitc-2020-000832supp006.pdf]

# Figure S5

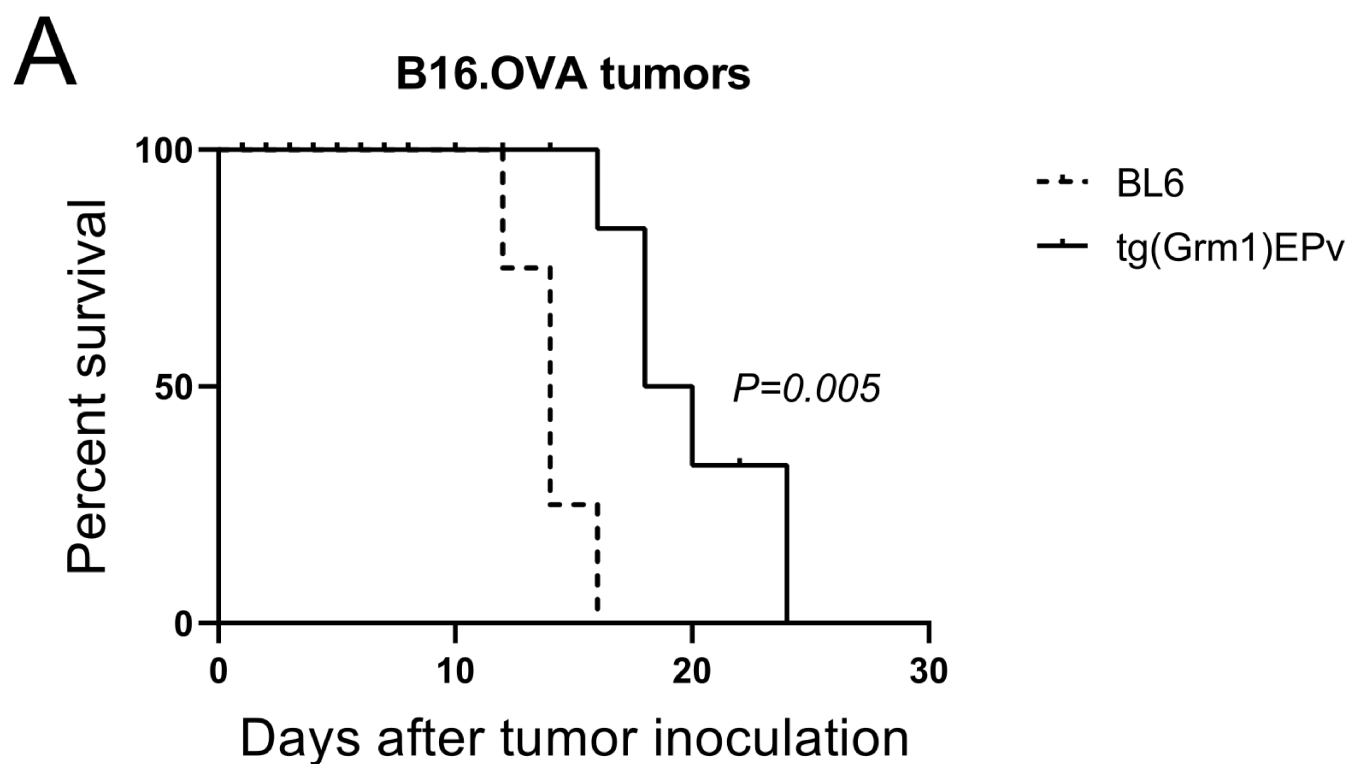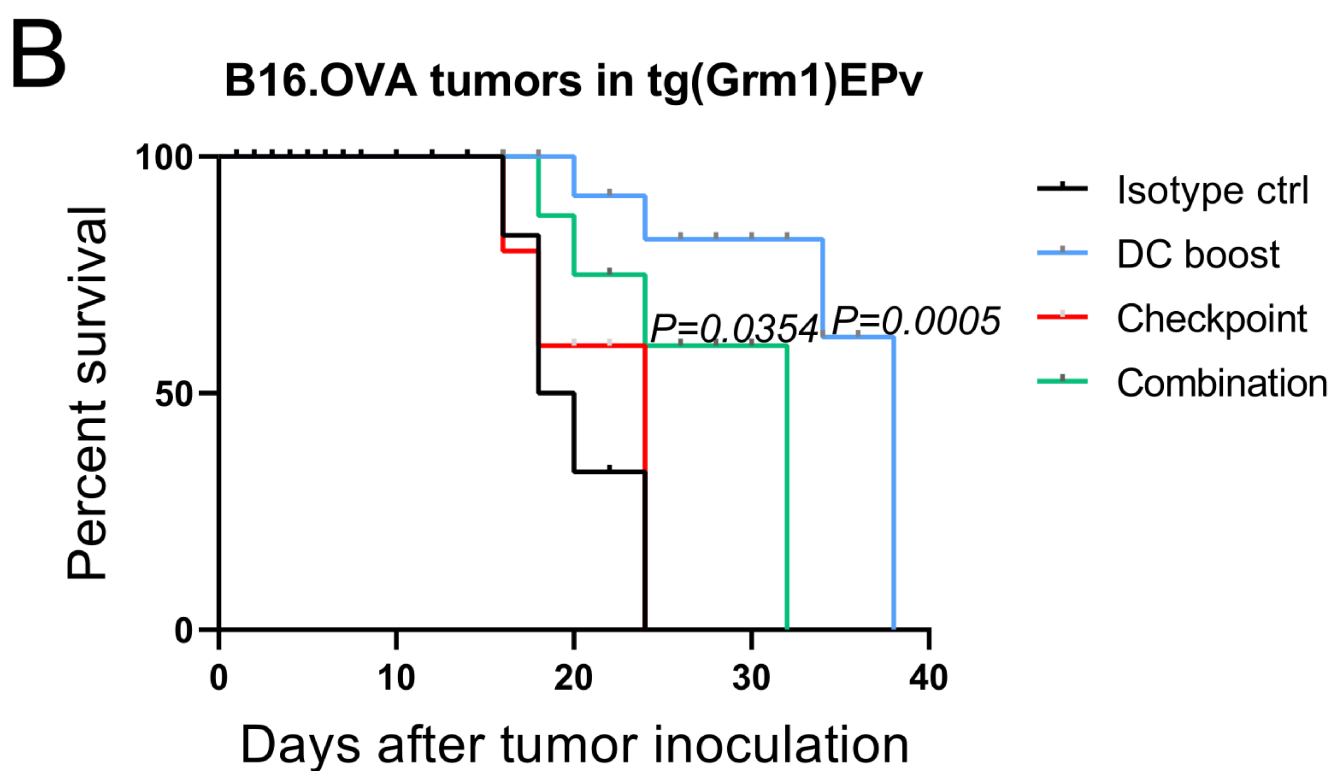

Supplement: Supplementary data [file jitc-2020-000832supp007.pdf]
