# Supplementary material for: Skin dendritic cells in melanoma are key for successful checkpoint blockade therapy
Source: J Immunother Cancer. 2021 Jan 6;9(1):e000832. doi: 10.1136/jitc-2020-000832 (PMC7789456; doi:10.1136/jitc-2020-000832)
Supplement: Supplementary data [file jitc-2020-000832supp003.pdf]

Supplementary Tables

Supplementary Table 1. Reagents used for *in vivo* experimental procedures

Reagent	mAb clones	Route of administration/ amount per mouse per injection	Company
Recombinant human Flt3 ligand	N/A	i.p./10 µg	Kindly provided by Tibor Keler, Celldex Therapeutics, Hampton, NJ, USA
Anti-mouse PD-L1 blocking mAb	MIH5	i.p./ 200 µg	Produced in-house
Anti-mouse PD-1 blocking mAb	RMP1-14	i.p./ 100 µg	Produced in-house
Anti-mouse TIM-3 blocking mAb	RMT3-23	i.p./ 100 µg	BioXcell, USA
Anti-mouse CD40 agonistic mAb	FGK45	i.d./25 µg	Produced in-house
Poly I:C	N/A	i.d./25 µg	Sigma-Aldrich
Anti-b-Gal rIgG1 mAb	GL113	i.d./1,6 µg	Produced in-house
Anti-b-Gal rIgG2a mAb	GL117	i.d./12,5 µg or i.p./200 µg	Produced in-house

Supplementary Table 2. Anti-mouse antibodies used for flow cytometry

Antibody	Clone	Company
CD45 FITC	30-F11	eBioscience
CD207 (Langerin) Alexa 488	929F3.01	Dendritics, Lyon, France
CD279 (PD-1) PE	RMP1-30	Biolegend
CD40 PE	3/23	BD Biosciences
CD274 (PD-L1) PE	MIH5	eBioscience
MHC-II PE	M5/114.15.2	eBioscience
CD207 (Langerin) PE	929F3.01	Produced in-house
FoxP3-PE	FJK-16s	eBioscience
MHC II PE/Dazzle 594	M5/114.15.2	Biolegend
CD3 PE/Dazzle 594	145-2C11	BD Biosciences
CD45 PerCP-Cy5.5	30-F11	BD Biosciences
CD3 PerCP-Cy5.5	17A2	Biolegend
CD8a PerCP-Cy5.5	53-6.7	Biolegend
CD3 PE-Cy7	17A2	Biolegend
CD279 (PD-1) PE-Cy7	RMP1-30	Biolegend
CD64-PE-Cy7	X54-5/7.1	Biolegend
CCR7-PE-Cy7	4B12	Biolegend
CD4 PE-Cy7	GK1.5	eBioscience
CD25 PE-Cy7	PC61	Biolegend
TIM-3 APC	RMT3-23	Biolegend
CD11b APC	M1/70	Biolegend
IFNg APC	XMG1.2	Biolegend
Ly6G APC	1A8	BD Biosciences
CD103 AF700	2E7	Biolegend
CD4 BV421	GK1.5	Biolegend
CD8a BV421	53-6.7	BD Biosciences
TNFa BV421	MP6-XT22	Biolegend
CD11c BV421	N418	Biolegend
CD103 BV421	2E7	Biolegend
Ly6C BV421	HK1.4	Biolegend
PD-1 BV421	RMPI-30	Biolegend
CD11c BV510	N418	Biolegend
CD103 BV510	2E7	Biolegend
CD4 BV510	RM4-5	Biolegend
CD8a BV510	53-6.7	Biolegend
CD90.1 BV510	OX-7	Biolegend

Supplementary Table 3. Primers used for RT-qPCR

**Gene id/protein**	**Code or sequence**	**Company**
Cd274/PD-L1	Mm00452054_m1	Thermofisher
Lgals9/Galectin-9	Mm00495295_m1	Thermofisher
Tnf/TNFa	Mm00443258_m1	Thermofisher
Cxcl9	Mm00434946_m1	Thermofisher
Cxcl10	Mm00445235_m1	Thermofisher
Gzmb	Mm00442834_m1	Thermofisher
Ifng	Mm01168134_m1	Thermofisher
TATA-binding protein	Forward: ACTTCGTGCAAGAAATGCTGAA Reverse: TGTCCGTGGCTCTCTTATTCTCA	Microsynth
