# Supplementary material for: Skin dendritic cells in melanoma are key for successful checkpoint blockade therapy
Source: J Immunother Cancer. 2021 Jan 6;9(1):e000832. doi: 10.1136/jitc-2020-000832 (PMC7789456; doi:10.1136/jitc-2020-000832)

Skin dendritic cells in melanoma are key for successful checkpoint blockade therapy

Anastasia Prokopi[1], Christoph H. Tripp[1], Bart Tummers[2], Florian Hornsteiner[1], Sarah Spoeck[1], Jeremy Chase Crawford[2], Derek R. Clements[3], Mirjana Efremova[4], Katharina Hutter[1], Lydia Bellmann[1], Giuseppe Cappellano[1], Bruno L. Cadilha[5], Sebastian Kobold[5], Louis Boon[6], Daniela Ortner[1], Zlatko Trajanoski[4], Suzie Chen[7], Tanja D. de Gruijl[8], Juliana Idoyaga[3], Douglas R. Green[2] and Patrizia Stoitzner[1,*]

- Melanoma progression is accompanied by a decrease in skin cDC2 and upregulation of PD-L1 and Galectin-9 in the tumor microenvironment.
- Responsiveness to checkpoint blockade can be restored by boosting DC function and activation in the tumors.
- Upon DC boost therapy, migratory skin cDC1 and cDC2 can prime melanoma-specific CD8+ T cells in the tumor-draining lymph nodes.

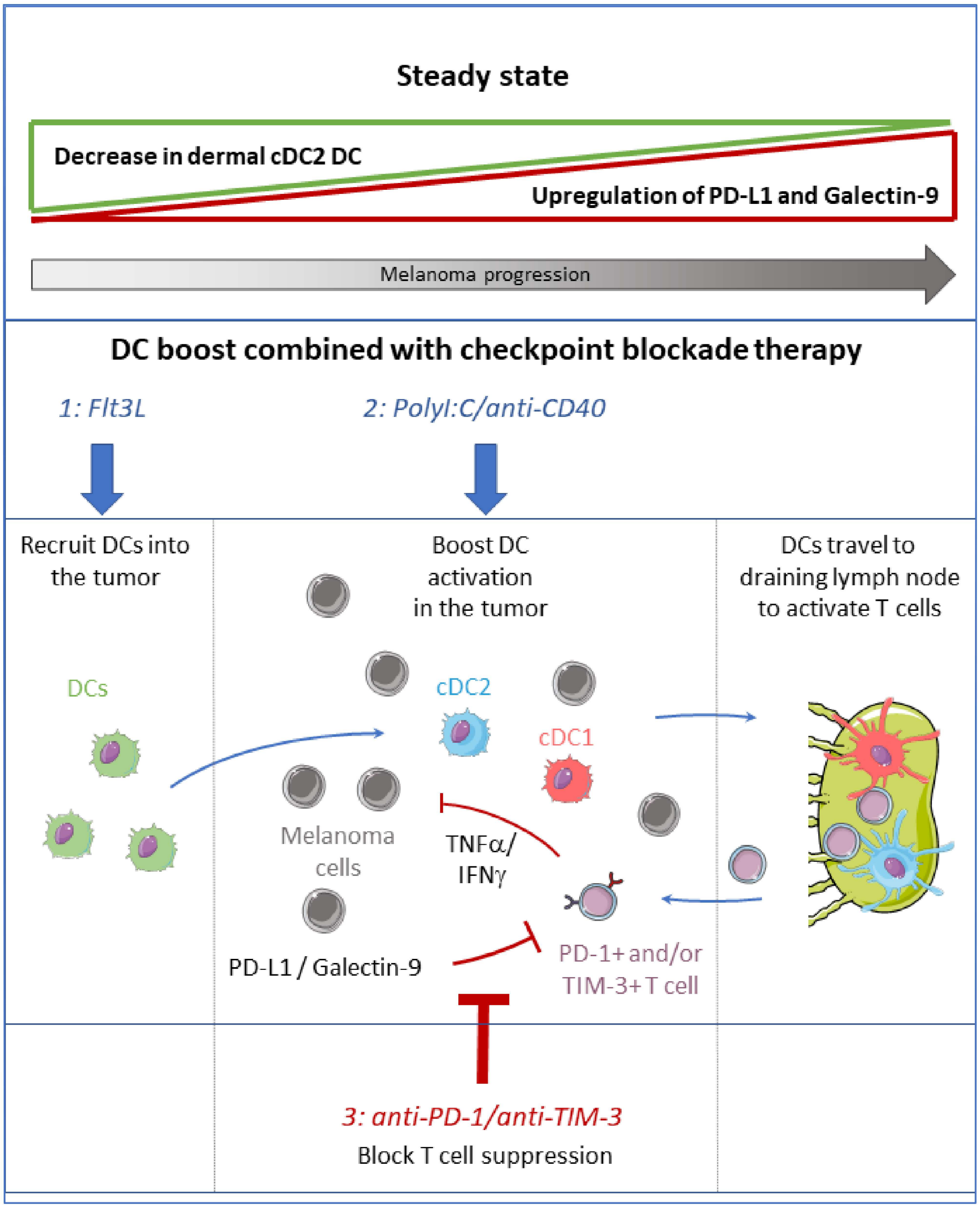

Supplement: Supplementary data [file jitc-2020-000832supp008.pdf]
